# Super-complexes of adhesion GPCRs and neural guidance receptors

**DOI:** 10.1038/ncomms11184

**Published:** 2016-04-19

**Authors:** Verity A. Jackson, Shahid Mehmood, Matthieu Chavent, Pietro Roversi, Maria Carrasquero, Daniel del Toro, Goenuel Seyit-Bremer, Fanomezana M. Ranaivoson, Davide Comoletti, Mark S. P. Sansom, Carol V. Robinson, Rüdiger Klein, Elena Seiradake

**Affiliations:** 1 Department of Biochemistry, Oxford University, Oxford OX1 3QU, UK; 2 Department of Chemistry, University of Oxford, Oxford OX1 3QZ, UK; 3 Max-Planck Institute of Neurobiology, Am Klopferspitz 18, 82152 Munich-Martinsried, Germany; 4 Child Health Institute of New Jersey, Robert Wood Johnson Medical School, Rutgers University, New Brunswick, New Jersey 08901, USA; 5 Department of Neuroscience and Cell Biology, Rutgers University, New Brunswick, New Jersey 08901, USA; 6 Department of Pediatrics, Robert Wood Johnson Medical School, Rutgers University, New Brunswick, New Jersey 08901, USA; 7 Munich Cluster for Systems Neurology (SyNergy), Feodor-Lynen-Straβe 17, 81377 Munich, Germany

## Abstract

Latrophilin adhesion-GPCRs (Lphn1–3 or ADGRL1–3) and Unc5 cell guidance receptors (Unc5A–D) interact with FLRT proteins (FLRT1–3), thereby promoting cell adhesion and repulsion, respectively. How the three proteins interact and function simultaneously is poorly understood. We show that Unc5D interacts with FLRT2 in *cis*, controlling cell adhesion in response to externally presented Lphn3. The ectodomains of the three proteins bind cooperatively. Crystal structures of the ternary complex formed by the extracellular domains reveal that Lphn3 dimerizes when bound to FLRT2:Unc5, resulting in a stoichiometry of 1:1:2 (FLRT2:Unc5D:Lphn3). This 1:1:2 complex further dimerizes to form a larger ‘super-complex' (2:2:4), using a previously undescribed binding motif in the Unc5D TSP1 domain. Molecular dynamics simulations, point-directed mutagenesis and mass spectrometry demonstrate the stability and molecular properties of these complexes. Our data exemplify how receptors increase their functional repertoire by forming different context-dependent higher-order complexes.

## Abstract (web summary)

FLRT proteins are known to interact with Lphns and Unc5s, mediating cell adhesion and repulsion respectively. Here the authors use crystallography, native mass spectrometry, molecular dynamics simulations and cell-based assays to show that these three proteins form large super-complexes with functions distinct from their smaller subcomplexes.

## Graphical abstract

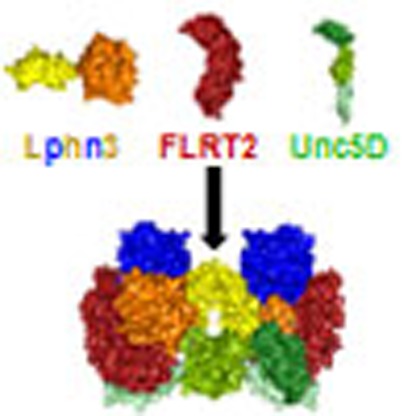

Brain development relies on a limited number of highly multifunctional cell guidance receptors that direct biological processes in a context-dependent way. Understanding how different receptors synergize in their effects is key to understanding the molecular mechanisms that control cell migration and neural wiring. Here we focus on three structurally distinct cell surface receptors: the fibronectin-leucine-rich transmembrane (FLRT) proteins, the Latrophilins (ADGRL, Lphn, Lec) and the uncoordinated-5 (Unc5 and Unc-5) receptors.

FLRTs are widely expressed in vertebrates and have recently emerged as powerful guidance factors in vascular, neural and early embryonic development^1^,^2^,^3^,^4^,^5^,^6^,^7^,^8^,^9^,^10^,^11^. The domain composition of FLRT is conserved across the three mammalian homologues (FLRT1–3) including an N-terminal extracellular leucine-rich repeat (LRR) domain, followed by a ∼60-residue linker, a fibronectin-like III domain, a single spanning transmembrane helix and a ∼95-residue intracellular C-terminal domain (Fig. 1a). The ectodomains of FLRTs contain a protease cleavage site and can be released from the expressing cell^4^. Most protein–protein interactions shown for FLRT involve the LRR domain, which promotes cell adhesion via interaction with itself (homophilic)^2^ or with Latrophilin^7^, and triggers cell repulsion by binding Unc5 receptors^1^,^4^,^5^. FLRT–FLRT and FLRT–Latrophilin interactions are mediated by overlapping binding sites on the concave face of the LRR^6^,^12^,^13^. Repulsive FLRT–Unc5 interactions are mediated via a distinct binding site at a lateral side of the FLRT LRR^5^. The adhesive and repulsive functions of the FLRT LRR are required during cortical development where they control the lateral and radial migration of pyramidal neurons, respectively^4^,^5^. Repulsive Unc5–FLRT interaction also plays a role in controlling the vascularization of the murine retina^5^ and in controlling neuronal laminar targeting in the inner plexiform layer^14^. In addition, FLRTs also bind fibroblast growth factor receptors via an interaction that requires the FLRT fibronectin-like domain, but the *in vivo* relevance of this interaction is unclear^11^.

Unc5 receptors were first discovered in invertebrates where they cause cell repulsion in response to netrin ligands^15^. Vertebrates express four homologues (Unc5A–D) that signal netrin-dependent cell repulsion and also act as dependence receptors^16^. Unc5 receptor expression is strongly suppressed in most cancers^17^,^18^, presumably due to pro-apoptotic and anti-angiogenic properties of Unc5 signalling^5^,^18^,^19^,^20^,^21^. Unc5 is also linked to late-onset Alzheimer's in humans^22^. The domain organization of Unc5 is generally conserved across species. The ectodomain consists of two N-terminal immunoglobulin-like (Ig) domains and two thrombospondin-like (TSP) domains. A transmembrane helix leads into the intracellular region including ZU5 and UPA domains, and a death domain (Fig. 1a). The two Ig and TSP1 domains of Unc5A (ref. 5), and most of the intracellular region of Unc5B (ref. 19) have been structurally characterized. The Ig1 domain is sufficient for binding to FLRT LRR proteins^5^.

Latrophilins are adhesion G-protein-coupled receptors (adhesion GPCRs) and known receptors of α-latrotoxin, a neurotoxic component of black widow spider venom. Deficient Latrophilin3 expression is associated with attention-deficit hyperactivity disorder in humans^23^,^24^ and restless behaviour in flies^25^. Vertebrate Latrophilin contains a ∼100-kDa ectodomain comprising an N-terminal lectin domain (Lec), also termed rhamnose-binding lectin-like domain, an olfactomedin-like domain (Olf), a glycosylated ∼100-residue linker, and a Horm/GPCR autoproteolysis-inducing (GAIN) domain containing an autoproteolysis motif that is conserved across adhesion GPCRs (Fig. 1a). The Lec, Olf and Horm/GAIN domains have been structurally characterized^6^,^26^,^27^. Endogenous ligands of vertebrate Latrophilins include FLRTs, neurexins and teneurins, which bind N-terminal domains of Latrophilin^7^,^28^,^29^. The interaction of these ligands with Latrophilin is best understood in the context of *trans*-cellular adhesion^7^,^28^,^30^. Using mutagenesis, we recently mapped the FLRT-binding site on the Latrophilin Olf domain^6^, which was further defined by recent structural analysis of dimers formed by FLRT3 LRR and Latrophilin3 Olf (refs 12, 13). Interestingly, a cell-based binding assay suggested that Latrophilin, FLRT and Unc5 form a ternary complex^13^, although the structural arrangement of the ternary interaction remained elusive.

In addition to a synaptic role, Latrophilins have also been shown to regulate mechanosensation^31^,^32^,^33^, cell polarization and cell migration in both vertebrates and invertebrates. In chicken, Latrophilin-2 has been identified as a regulator of the epithelial–mesenchymal transition, the process by which polarized epithelial cells assume a mesenchymal phenotype, with enhanced migratory and invasive capacity^34^. In *Caenorhabditis elegans*, maternal and zygotic expression of the Latrophilin orthologue (*lat-1)* is essential for robust establishment of anterior–posterior tissue. *C. elegans lat-1* mutants also display defects in the division plane alignment of epidermal seam cells, leading to defects in seam cell migration^35^. Like many other GPCRs, mutations in Lphns are associated with multiple types of human cancer^36^. How the binding of Latrophilin to extracellular ligands impacts on cell migration is still poorly understood. We recently showed that Latrophilin-binding triggers an adhesive response in FLRT-expressing HeLa cells and a cell repulsive response in cortical neurons^6^, suggesting that Latrophilin is able to act as a bifunctional protein.

Here we show that co-expression of Unc5D in FLRT2-expressing cells reduces the adhesion of these cells in response to external Latrophilin3 protein. The data point to an anti-adhesive role for Unc5D, which requires direct interaction with FLRT2/Latrophilin3. In agreement with these results, we show binding between FLRT2, Unc5D and Latrophilin3 proteins in solution and at the surface of cells. We find that while FLRT2–Latrophilin3 and FLRT2–Unc5D complexes consist of 1:1 dimers, complexes of FLRT2, Unc5D and Latrophilin3 ectodomains form large assemblies containing two copies of Latrophilin3 for each copy of FLRT2 and Unc5D. We combine molecular dynamics simulations with mass spectrometry (MS) to characterize the protein–protein binding surfaces that give rise to these assemblies. Structure-based site-directed mutagenesis allows us to break the complexes down into specific smaller subunits. Taken together, the data we present here reveal unexpected large complexes of FLRT, Latrophilin and Unc5, and first insights into how these three-protein complexes are functionally distinct from their smaller subcomponents.

## Results

### Unc5D controls Latrophilin3–FLRT2-mediated cell adhesion

**Figure 1:**
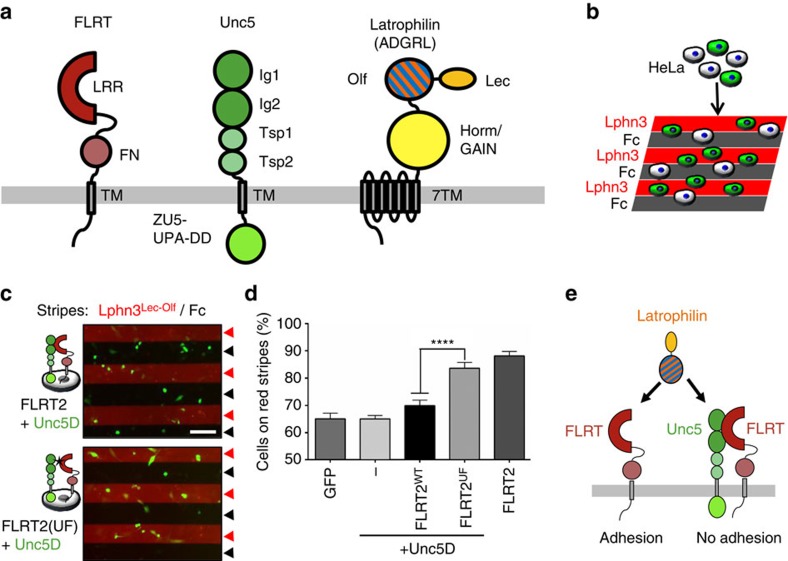
Unc5D attenuates Lphn3-FLRT2-mediated adhesion. (**a**) The domain composition of FLRT proteins, Unc5 receptors and Latrophilins is highly conserved across vertebrates. Domains are coloured according to protein type: FLRT (reds); Unc5 (greens); and Latrophilin (yellow/orange or blue). Linkers of unknown fold are shown as black lines. (**b**) We performed stripe assays in which transfected HeLa cells were seeded on alternating stripes of Lphn3 protein or control Fc protein. (**c**) Stripe assays in which HeLa cells (green) expressing GFP control, Unc5D alone, FLRT2 alone, FLRT2+Unc5D or FLRT2(UF)+Unc5D were seeded on alternating stripes of Lphn3^Lec–Olf^ (red arrowheads) or control Fc protein (black arrowheads). FLRT2(UF) is a FLRT2 mutant that is unable to bind Unc5D via the ectodomain^5^. Scale bar, 100 μm. (**d**) The percentage of transfected cells adhering to red (Lphn3^Lec–Olf^) stripes was quantified by measuring the fraction of green pixels present on red stripes in each image. A value of 100% would represent an image where all transfected cells have adhered to red stripes. Statistical significance was determined using an unpaired, two-tailed *t*-test (****P<0.0001). Error bars represent the s.e.m. and results are averaged over seven repeat experiments performed in duplicate. (**e**) Summary cartoon showing that Latrophilin–FLRT interaction causes cell adhesion, however in *cis* interaction of FLRT and Unc5 attenuates Latrophilin-induced adhesion.

We performed stripe assays essentially as previously described^6^, by seeding transfected HeLa cells on alternating stripes of immobilized mouse Latrophilin3 Lec+Olf (Lphn3^Lec–Olf^) or Fc control protein, which does not elicit any adhesive or repulsive cell response (Fig. 1b). The FLRT^LRR^–Lphn3^Olf^ interaction is adhesive^6^, and so FLRT2-transfected HeLa cells adhere strongly (>80% of cells) to Lphn3^Lec–Olf^ stripes (Fig. 1d). Here we show that double-transfected HeLa cells expressing FLRT2 and Unc5D adhere significantly less (∼70% of cells) to Lphn3^Lec–Olf^, similar to control cells or cells transfected with only Unc5D (Fig. 1c,d). We hypothesized that Unc5D may be able to control FLRT2-dependent adhesion by interacting with FLRT2 in *cis*. We used a previously characterized FLRT2 mutant (FLRT2(UF)), which is unable to bind Unc5 via the ectodomain^5^, but still binds Lphn3^Lec–Olf^ (ref. 6). Coexpression of FLRT2(UF) with Unc5D switched the cell response back to >80% adhesion (Fig. 1c,d). These data lead to a model in which Unc5D acts as a switch that attenuates the adhesive effect of Latrophilin3 on FLRT2-expressing cells (Fig. 1e).

### FLRT mediates binding between Latrophilin and Unc5

**Figure 2:**
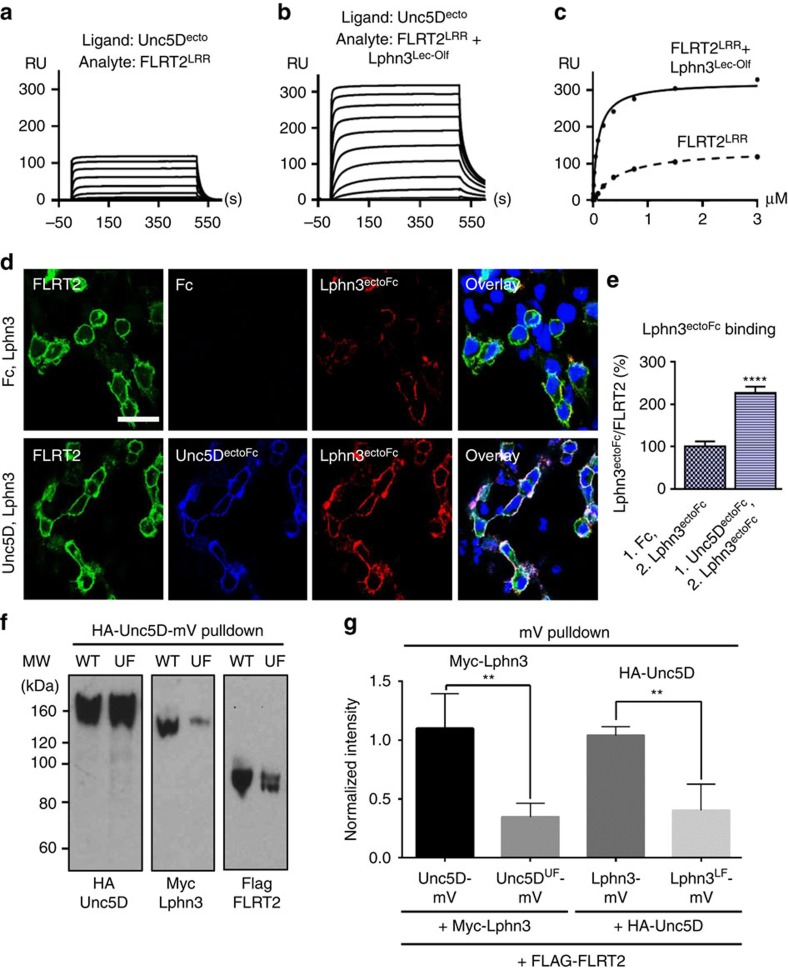
FLRT2 binds cooperatively to Unc5D and Lphn3. (**a**,**b**) Unc5D^ecto^ was immobilized on the surface of a streptavidin-coated CM5 chip and different concentrations of FLRT2^LRR^ (**a**) or a 1:2 molar ratio of FLRT2^LRR^+Lphn3^Lec–Olf^ (**b**) injected as analytes. (**c**) The data in **a** and **b** were fitted using 1:1 and 1:(1:1) binding models. The presence of the Lphn3^Lec–Olf^ enhances the apparent affinity of the Unc5D^ecto^-FLRT2^LRR^ interaction by ∼5-fold (the apparent *K*_d_s change from 460 to 94 nM). Note that subsequent experiments show that FLRT2^LRR^, Unc5D^ecto^ and Lphn3^Lec–Olf^ do not bind in 1:1:1 stoichiometries, therefore the shown *K*_d_ values are indicative only. Additional data are available in Supplementary Fig. 1. (**d**) A HEK293 cell-based binding assay was used to test the binding of Lphn3^ectoFc^ protein (red) to FLRT2-expressing cells (green). Cells were previously incubated with Fc control protein or Unc5D^ectoFc^ protein (blue). Scale bar, 30 μm. (**e**) Quantification of the assay presented in **d**. The ratio of Lphn3^ectoFc^/FLRT2 (red/green signal) was quantified and plotted. Lphn3^ectoFc^ bound after incubation with Fc (left bar) was used as reference (100%). The data show that Lphn3^ectoFc^ binds better to FLRT2-expressing cells that were previously incubated with Unc5D^ectoFc^, compared with the control cells that were previously incubated with Fc control protein. Statistical significance was determined using an unpaired, two-tailed *t*-test (****P<0.0001). A total of 15 images from 2 separate experiments were analysed per condition. (**f**) mV-fused proteins were pulled down from lysate of HEK293 cells transfected with HA-Unc5D-mV, Myc-Lphn3 and FLAG-FLRT2. Blots revealed that wild-type Unc5D pulls down FLRT2 and Lphn3 more efficiently than the non-FLRT-binding mutant Unc5D^UF^. The reverse experiment using wild-type or mutant Myc-Lphn3-mV and HA-Unc5D gave equivalent results. (**g**) Quantification of the experiment shown in **f** and the reverse Myc-Lphn3-mV pull-down experiment. Results were averaged over 3–4 independent experiments. Statistical significance was determined using an unpaired, two-tailed *t*-test (**P=0.0031 and **P=0.0053, for the Unc5D and Lphn3 pull downs, respectively). All error bars represent the s.e.m.

To test for formation of a ternary complex of Unc5D, FLRT2 and Latrophilin3, we performed surface plasmon resonance experiments using purified mouse FLRT2 LRR domain (FLRT2^LRR^), rat Unc5D ectodomain (Unc5D^ecto^) and mouse Lphn3^Lec–Olf^. The data indicate that Unc5 and Latrophilin3 bind to FLRT2 simultaneously, and that the resulting complex is of high affinity (Fig. 2a,b). Assuming a simple 1:1:1 binding model (Fig. 2c) for the three proteins suggests a >5-fold increase in affinity for Unc5D^ecto^ binding to FLRT2^LRR^+Lphn3^Lec–Olf^ compared with just FLRT2^LRR^ alone. We obtained consistent results with isothermal titration calorimetry (ITC) experiments using the purified ectodomains of mouse FLRT2 (FLRT2^ecto^), rat Unc5D^ecto^ and the Olf domain of mouse Latrophilin3 (Lphn3^Olf^; Supplementary Fig. 1). In a control experiment we show that Unc5D does not bind Lphn3^Olf^ in the absence of FLRT2^ecto^ (Supplementary Fig. 1). We used a protein overlay assay to test binding of Fc-tagged Lphn3 and Unc5D ectodomains to full-length FLRT2 expressed in HEK293 cells, to verify that ternary complex formation can also occur on the surface of cells. Cells over-expressing transmembrane FLRT2 bound significantly more Lphn3 ectodomain when applied after incubation with Unc5D ectodomain, also suggesting cooperative binding (Fig. 2d,e). Pull-down data using murine brain lysates are in agreement with the ternary complex forming *in vivo* (Supplementary Fig. 2). To verify that full-length cell surface Lphn3, FLRT2 and Unc5D form a ternary complex, we performed an anti-GFP pull-down from lysate of cells transfected with a full-length HA-Unc5D mono-Venus (mV) fusion protein, Myc-Lphn3 and FLAG-FLRT2, showing that full-length Unc5D can pull down both Lphn3 and FLRT2 (Fig. 2f). This effect is significantly reduced when wild-type Unc5D is replaced with our previously characterized non-FLRT-binding mutant Unc5D^UF^, showing that high-affinity complex formation is mediated by FLRT2 and depends on interactions via the ectodomains (Fig. 2f,g). The reverse experiment, in which mV-tagged Lphn3 (and its non-FLRT-binding mutant Lphn3^LF^ (ref. 6)) was used to pull down Unc5D through FLRT2, yielded equivalent results further suggesting that FLRT2 mediates Lphn3/FLRT2/Unc5D complex formation in HEK293 cells (Fig. 2g).

### Structure of the FLRT2^LRR^ Unc5D^Ig^ Lphn3^Lec-Olf^ tetramer

**Figure 3:**
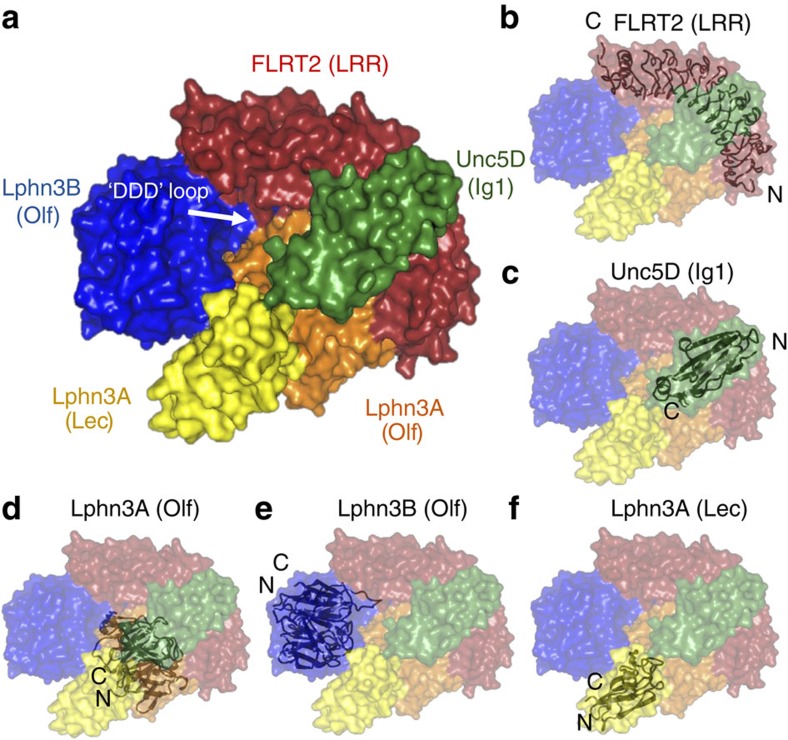
Crystal structure of the tetrameric complex of FLRT2^LRR^ and Unc5D^Ig^ and Lphn3^Lec-Olf^. (**a**) The protein chains in the structure are coloured FLRT2^LRR^ (red), Unc5D^Ig^ (green), Lphn3A^Lec–Olf^ (Olf: orange, Lec: yellow), Lphn3B^Lec–Olf^ (Olf: blue). The Lec domain of Lphn3B is not resolved in the crystal structure, and is presumably flexible within the crystal. The location of the DDD loop in Lphn3B is indicated. (**b**–**f**) Individual domains are shown as black cartoons within the surface model of the complex. The locations of N and C termini are indicated. See also Supplementary Figs 3 and 4.

We expressed and purified mouse Lphn3^Lec-Olf^, mouse FLRT2^LRR^ and rat Unc5D Ig domain 1 (Unc5D^Ig^) using HEK293 cells^37^, mixed the proteins in a 1:1:1 molar ratio and crystallized the resulting complex. The crystals diffracted up to 6 Å maximum resolution (Table 1). We performed molecular replacement with the individual components, previously solved at higher resolution^5^,^6^, using PHASER (ref. 58), and obtained high-quality electron density maps and reliable signal for all chains despite the low resolution (Supplementary Fig. 3). The resulting structure revealed an unexpected stoichiometry, in which each copy of FLRT2^LRR^ binds one copy of Unc5D^Ig^ and two copies of Lphn3^Lec–Olf^ (Fig. 3a–f). Each asymmetric unit contains three such 1:1:2 ‘tetramers' (Supplementary Fig. 3a). Within each tetramer, Unc5D^Ig^ is bound to FLRT2^LRR^ in the same orientation as the 1:1 complex structure we solved previously (root mean squared deviation is 0.7 Å for 423 aligned Cα atoms)^5^. The Olf domain of one Lphn3^Lec–Olf^ molecule (Lphn3A^Lec–Olf^) occupies a binding site on the concave surface of FLRT2^LRR^ (Fig. 3a,d), which has previously been identified as the FLRT2 dimerization surface^5^. The arrangement is in close agreement with our previous mutagenesis data^6^, and the corresponding interface recently revealed in 1:1 complex crystal structures of FLRT3^LRR^ and Lphn3^Olf^ (refs 12, 13). This mode of interaction between an LRR protein and a globular domain is also found in other synaptic complexes, for example, the netrin-G ligand/netrin-G complex^38^. Comparison of Lphn3^Lec–Olf^ to the unliganded structure of Lphn3^Lec–Olf^ (ref. 6) reveals a reorientation of the lectin domain to make contacts with Unc5D Ig/Ig2 and the Lphn3A Olf domain (Supplementary Fig. 4). The Olf domain of the second copy of Lphn3^Lec–Olf^ (labelled Lphn3B^Lec–Olf^) makes extensive contacts with the Olf and Lec domains of Lphn3A^Lec–Olf^ (Fig. 3a,e) and inserts a negatively charged loop (S393-N405) into a cleft formed by FLRT2^LRR^ and Lphn3A^Lec–Olf^. We termed this loop the ‘DDD loop' (Fig. 3a) as it contains three consecutive aspartate residues: D397, D398 and D399. The DDD loop was unresolved in our previous high-resolution crystal structures of Lphn3^Lec–Olf^ alone^6^,^12^, suggesting that it only becomes ordered on engagement with FLRT2^LRR^/Lphn3A as found in the complex. The lectin domain of Lphn3B^Lec–Olf^ makes no contacts with the complex and its electron density is not visible in the map.

**Figure 4:**
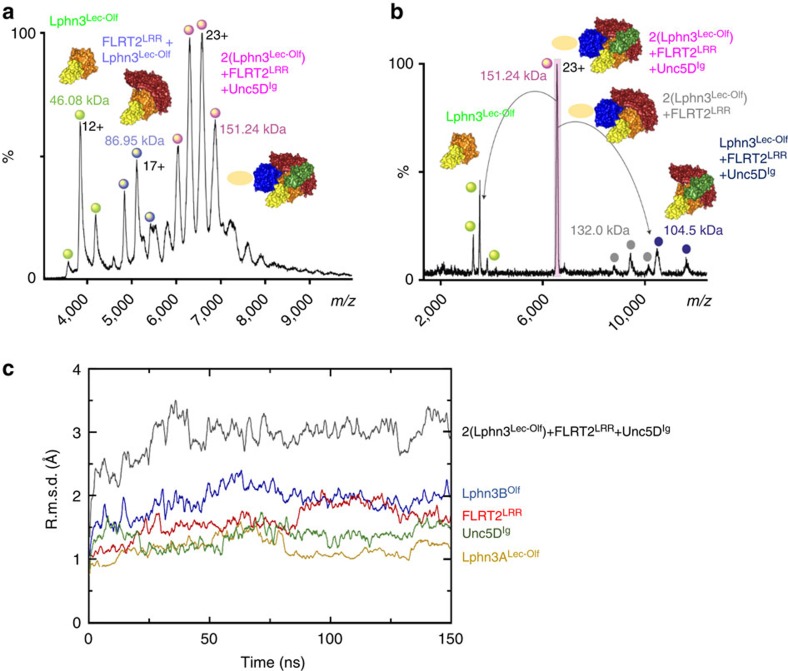
Mass spectrometry and molecular dynamics simulations demonstrate the 1:1:2 complex forms in solution. (**a**) We mixed purified FLRT2^LRR^, Unc5D^Ig^ and Lphn3^Lec–Olf^ in a 1:1:2 ratio and acquired MS data. The profile reveals masses corresponding to a 1:1:2 tetramer, as well as smaller subcomplexes and Lphn3^Lec–Olf^ on its own. The assigned species are depicted as surface models above the corresponding peaks. Colours of the models are as in Fig. 3. The Lphn3B Lec domain, which is not resolved in the crystal structure, is shown as a pale yellow oval. (**b**) We isolated the tetramer peak shown in **a** and performed MS/MS to reveal subcomplexes present in the original peak species. (**c**) The 1:1:2 tetramer model remained stable over 150 ns of molecular dynamics simulation. Cα root mean squared deviations (r.m.s.d.s) across the trajectories are plotted for the total complex (black) and for the individual subcomponents (colours as indicated).

We used MS and molecular dynamics simulations to assess whether the tetrameric arrangement found in the crystal also exists in solution. For MS, we mixed purified FLRT2^LRR^, Unc5D^Ig^ and Lphn3^Lec–Olf^ in a 1:1:2 molecular ratio and injected the proteins at concentrations of ∼1.5 mg ml^−1^. The resulting spectra revealed masses corresponding to a 1:1:2 tetramer (151.24 kDa), as well as smaller subcomponents: 86.95 kDa, consistent with a 1:1 Lphn3^Lec–Olf^:FLRT2^LRR^ complex and 46.08 kDa, consistent with Lphn3^Lec–Olf^ on its own (Fig. 4a). We performed tandem MS (MS/MS) of the 151.24-kDa peak to validate the complex composition. This experiment resulted in peaks of 132.0 kDa (consistent with a 2:1 Lphn3^Lec–Olf^:FLRT2^LRR^ complex) and 104.5 kDa (consistent with a 1:1:1 complex of Lphn3^Lec–Olf^:FLRT2^LRR^:Unc5D^Ig^), suggesting that one of the two Lphn3^Lec–Olf^ chains, presumably Lphn3B^Lec–Olf^, is more weakly bound than the other (Fig. 4b). During molecular dynamics simulations of the entire tetrameric complex, only modest overall displacement was observed for the individual chains as well as globally, further suggesting that the complex is conformationally stable (Fig. 4c).

### Structure of the FLRT2^LRR^ Unc5D^IgIgTsp^ Lphn3^Lec–Olf^ octamer

**Figure 5:**
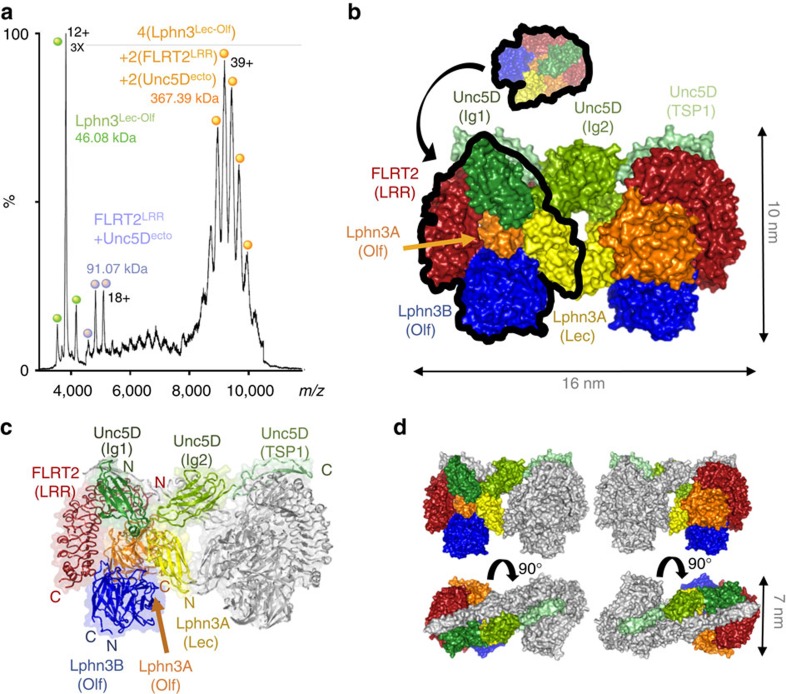
Crystal structure of the octameric complex of FLRT2^LRR^ and Unc5D^IgIgTSP^ and Lphn3^Lec–Olf^. (**a**) MS reveals that FLRT2^LRR^, Unc5D^ecto^ and Lphn3^Lec–Olf^ form a large oligomer, 2 × the size of a putative 1:1:2 complex. (**b**) The crystal structure of FLRT2^LRR^, Unc5D^IgIgTSP^ and Lphn3^Lec-Olf^ reveals a pseudo-symmetric molecule in which two copies of the 1:1:2 tetramer described in Fig. 3 are brought together by antiparallel packing of the Unc5D domains Ig2 and TSP1. The proteins are coloured FLRT2^LRR^ (red), Unc5D^IgIgTSP^ (Ig1: dark green; Ig2: medium green; TSP1: pale green), Lphn3A^Lec–Olf^ (Olf: orange; Lec; yellow), Lphn3B^Lec–Olf^ (Olf: blue). The Lec domain of Lphn3B is not resolved in the crystal structure, and is presumably flexible within the crystal. (**c**) Cartoon view. One-half of the pseudo-symmetric molecule is shaded in grey. The other colours are as in **b**. (**d**) Surface views with colours as in **c**.

On complexation of FLRT2^LRR^ and Lphn3^Lec–Olf^ with a longer construct of Unc5D, which comprises all of the Unc5D extracellular domains (Unc5D^ecto^), MS analysis revealed masses (367.39 kDa) that were twice as large as those expected for the 1:1:2 tetramer, suggesting dimerization of the tetramer into an octamer (Fig. 5a). Using Unc5D constructs of different length, we performed multi-angle light scattering (MALS) experiments to test which regions within Unc5D^ecto^ are responsible for the formation of this larger oligomer. We found that the first TSP domain (TSP1) of Unc5D is required for octamer formation (Supplementary Fig. 5a). This high-affinity octamer requires the presence of all three proteins. The two-protein complexes Lphn3^Lec-Olf^+FLRT2^LRR^ and Unc5D^ecto^+FLRT2^LRR^ result in masses corresponding to 1:1 dimers, Unc5D^ecto^ alone runs as a monomer (Supplementary Fig. 5). On the basis of these results, we carried out crystallization trials using Lphn3^Lec–Olf^, FLRT2^LRR^ and a construct comprising the two Ig and the TSP1 domains of Unc5D (Unc5D^IgIgTSP^). The resulting crystals diffracted to 3.4 Å maximum resolution (Table 1). We determined the structure of the octamer by molecular replacement using the tetrameric model described above and a homology model of Unc5D^IgIgTSP^, generated with the SWISS-MODEL server^39^, based on the structure of the Unc5A ectodomain^5^. The crystal structure reveals that the larger oligomer observed indeed forms through dimerization of the tetramer described above (Fig. 5b). Two chains of Unc5D^IgIgTSP^ (now including the extra domains Ig2 and TSP1) pack into an antiparallel arrangement, providing a bridge between the two pseudo-symmetric halves of the complex (Fig. 5c,d). Unc5D TSP1 is in contact with the lateral side of the FLRT2^LRR^, adjacent to the binding site for Unc5D Ig1. As in the tetrameric structure, the Lec domain of Lphn3B^Lec–Olf^ points to a solvent channel and is disordered. Compared with the Unc5A^ecto^ structure (PDB accession 4V2A), Unc5D^IgIgTSP^ bends at two hinge positions (Ig1–Ig2, Ig2–TSP1; Supplementary Fig. 6).

### Molecular architectures of novel protein–protein interfaces

**Figure 6:**
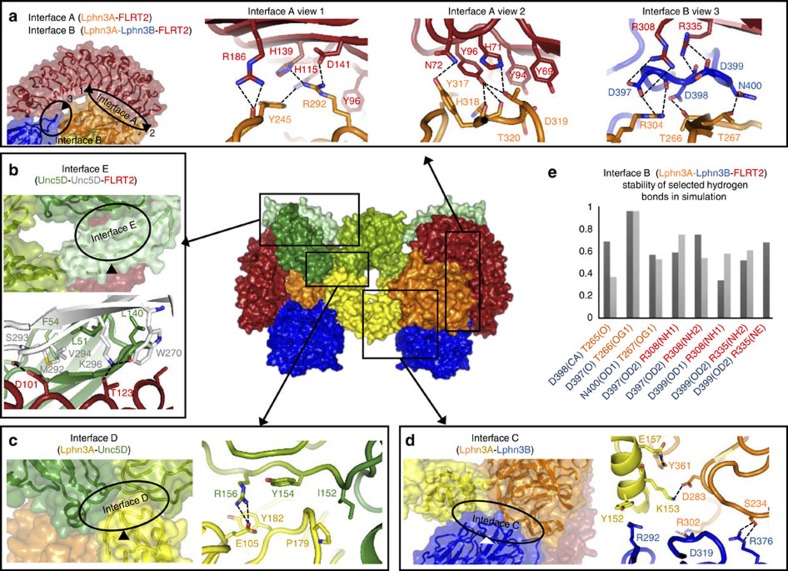
Protein–protein interactions within the super-complex. (**a**–**d**) Novel protein–protein binding surfaces (interfaces A–E) in the FLRT2^LRR^/Lphn3^Lec–Olf^/Unc5D^IgIgTSP^ complex are shown. Selected residues are shown as sticks. Selected putative hydrogen bonds are indicated as black dashed lines. (**e**) The stability of putative hydrogen bonds during molecular dynamics simulation is shown as the relative fraction of time they existed compared with the entire run time. The results are shown for interface B atoms within the two halves of the pseudo-symmetric complex (chains A–D: dark grey; chains E–F: light grey). Summaries of the main hydrogen bonding residues between the different protein chains are provided in Supplementary Table 1.

The limited resolution X-ray diffraction data we collected do not reveal detailed information on the atomic-level molecular interactions within the protein–protein binding surfaces. We therefore performed extensive molecular dynamics simulations to provide an improved model of the interacting surfaces and to produce detailed information on their hydrogen-bonding patterns (Supplementary Table 1). We describe here five novel protein–protein binding surfaces (termed interfaces A–E) that are found within the structures (Fig. 6a–d). Interface A provides contacts between the Lphn3A Olf domain and the concave surface of FLRT2^LRR^ (Fig. 6a). The protein surface buried (∼1,700 Å^2^) is rich in aromatic residues (Tyr and His). A number of hydrogen bonds are formed between the two surfaces (Fig. 6a and Supplementary Table 1), including a salt bridge between FLRT2 D141 and Lphn3A R292. Interface A is highly conserved between FLRT2 and FLRT3, revealing only minor sequence differences, such as FLRT2 H71 (Fig. 6a), which is replaced by a glutamine in FLRT3. Interface B includes the negatively charged DDD loop of Lphn3B, which binds to positively charged surfaces formed by FLRT2 and Lphn3A (Fig. 6a). Important hydrogen bonds in this interface are summarized in Fig. 6e. FLRT2 R308 and R335, and Lphn3A R304 provide charge complementarity and form salt bridges with Lphn3B D397, D398 and D399. Lphn3A threonines (T265, T266 and T267) provide additional hydrogen bonds to the DDD motif and the neighbouring N400 side chain. Charge complementarity between Lphn3B E401 and Lphn3A R263 further stabilize the Lphn3B DDD loop in its position. Interestingly, mutation of human Lphn2 R196 (the equivalent residue to R263 in murine Lphn3) has been identified in human cancer cases^36^. Lphn3B Olf further interacts with Lphn3A Olf and Lec domains in interface C (Fig. 6d). A salt bridge is formed between the Lphn3A Lec and Olf domains (involving K153 and D283), which presumably stabilizes the binding surface presented to Lphn3B. Binding of Lphn3B to FLRT2 and Lphn3A (interfaces B and C) buries a total surface of ∼2,400 Å^2^. Interface D is formed by the Lec domain of Lphn3A, which contacts a groove on the surface of Unc5D Ig1 and Ig2 (Fig. 6c), burying ∼1,200 Å^2^ total protein surface. A salt bridge forms between Lphn3A E105 and R156, located in the Unc5D Ig1–Ig2 linker. The interface also contains hydrophobic regions, for example, formed by Lphn3A P179 and Unc5D I152. Interface E (∼1,800 Å^2^ buried surface) includes contacts between the Unc5D TSP domain and a binding site formed by Unc5D Ig1 bound to FLRT2 LRR (Fig. 6b). This interface is observed only in the octamer structure. Important interactions are provided by K296 in the TSP1 domain, interacting with FLRT2 T123. A hydrophobic pocket is formed between Unc5D Ig1 and the TSP domain, involving also L51, F54 and M292. As well as contributing to the overall hydrophobicity of this pocket, Unc5D M292 may participate in a long-range interaction with F54 from the neighbouring Unc5D Ig1 domain, as previously shown for methionine/aromatic residues in other protein–protein interfaces^40^.

### Structural manipulation of novel super-complex interfaces

We were interested in producing mutants that would disrupt specific interfaces, allowing us to control the oligomerization state of the three proteins. Such mutants will be valuable tools for future functional analysis of FLRT/Lphn/Unc5 complexes. We previously published mutants that disrupt individual 1:1 interfaces formed by FLRT2:Unc5D (mutants UF)^5^ and Lphn3A:FLRT2 (mutants LF and FF)^6^. Here we target the novel interface B, which is formed on binding of the second copy of Lphn3, Lphn3B, to the complex. We produced two Lphn3^Lec–Olf^ mutant proteins: a DDD ‘charge-reversal mutant' (D397R, D398R and D399R) and a mutant containing an artificial N-linked glycosylation site at position 397 (D397N+D399T) in the DDD loop. We performed MS with Unc5D^Ig^ and FLRT2^LRR^ to assess the complexation abilities of these Lphn3^Lec–Olf^ mutants. In contrast to wild-type Lphn3^Lec–Olf^ (Fig. 4a), using the charge-reversal mutant resulted in peaks of ∼110 kDa appearing, corresponding to masses of a 1:1:1 trimeric complex. The amount of species assigned to the 1:1:2 tetramer was relatively reduced (Supplementary Fig. 7a). Introduction of an artificial N-linked glycan in the DDD loop more completely disrupted the formation of the tetrameric complex, presumably because the bulky glycan is sterically incompatible with Lphn3B binding in the conformation described here (Supplementary Fig. 7b). From herein we refer to this mutant as the Lphn3^Lec–Olf^ DDD mutant. Notably, both mutants still resulted in masses compatible with 1:1:1 trimeric complexes, demonstrating that the DDD loop is important for binding of Lphn3B^Lec–Olf^, but not for Lphn3A^Lec–Olf^. This is also consistent with surface plasmon resonance experiments in which immobilized Unc5D^ecto^ showed an intermediate level of binding to FLRT2^LRR^+Lphn3^Lec–Olf^ DDD mutant compared with FLRT2^LRR^+wild-type Lphn3^Lec–Olf^ or FLRT2^LRR^ alone (Supplementary Fig. 7c).

We were also interested in producing a mutant in interface E, which would disrupt the formation of the octameric complex, without impacting on the tetramer. Interface E involving the Unc5D TSP1 domain is necessary for the octamer formation, but not tetramer formation. Therefore, we introduced an N-linked glycosylation site at Unc5D M292 (M292N+V294T). MALS analysis confirmed that this mutation reduces the octamer to masses corresponding to the tetramer (Supplementary Fig. 7d).

## Discussion

The development of the nervous system requires a complex series of cell guidance events, which are directed by relatively few cell guidance receptors. Compensating for the relatively low number of receptors, is the ability of many receptors to produce distinct responses, depending on which molecules they interact with in their local environment. Such interactions are often highly dynamic, resulting in adhesion or repulsion of cells triggered by a combination of interacting receptors^41^,^42^.

Here we show how addition of a repulsive guidance receptor (Unc5D) to two adhesive proteins (FLRT2 and Latrophilin3) modulates the adhesive cell response via an indirect mechanism, without disrupting the FLRT–Lphn3 ectodomain interaction directly. We biophysically and structurally characterize the protein complexes formed by the extracellular domains of these proteins, revealing unexpectedly large assemblies (super-complexes) with unequal stoichiometries. These super-complexes contain two copies of Latrophilin for each copy of Unc5 and FLRT, suggesting that Latrophilin may act as a constitutive dimer or is dimerized by FLRT/Unc5 binding. Previous studies have shown that *C. elegans* Latrophilin ectodomains exist as a mixture of monomers and non-covalently linked dimers^43^. The authors suggested a mechanism for Latrophilin forward signalling in which ligand binding to the rhamnose-binding lectin domain of Latrophilins induces dimerization of ectodomains, leading to the cross activation of the transmembrane (7TM) domain by the partner molecule. Given our complex crystal structures, a similar mechanism may also occur in mammals. Ligand-induced dimerization or clustering at the cell surface has been shown for many classes of receptors, such as the epidermal growth factor receptors^44^, fibroblast growth factor, plexins^45^ and ephrin receptors^46^,^47^, which all dimerize or oligomerize on ligand binding. Despite the large number of structurally characterized 1:1 or 2:2 cell guidance receptor complexes, only few structures are known for complexes involving more than two proteins. Other examples include the repulsive guidance molecules, which act as a molecular bridge to form a 2:2:2 ternary complex with bone morphogenetic proteins and Neogenin (NEO1)^48^, and the 2:2:2 complex formed by plexin, semaphorin and neuropilin^49^. To the best of our knowledge, the 2:2:4 complex structure of FLRT2/Unc5D/Lphn3 we present here is the first example of a super-complex formed by three cell guidance receptors with this stoichiometry.

**Figure 7:**
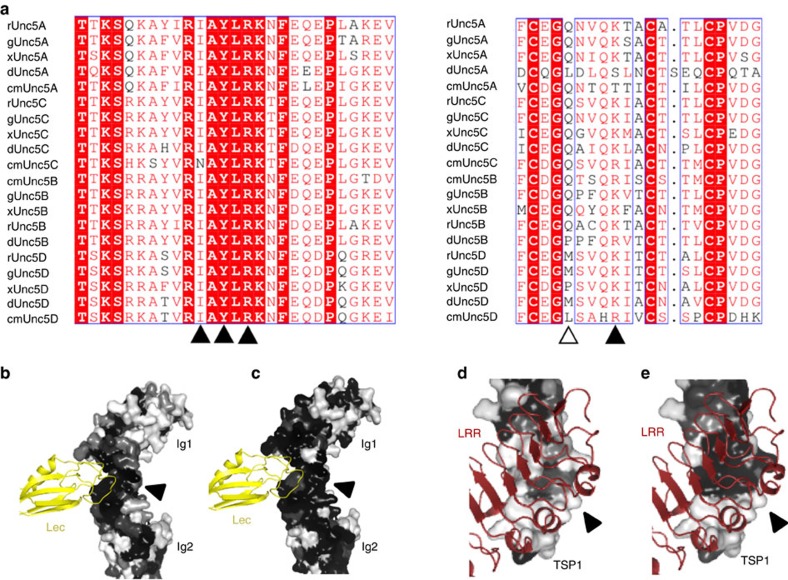
Sequence conservation across the Unc5 family of proteins. (**a**) Sequence alignments of Unc5A–D from rat (r), chick (g), frog (x), fish (d) and shark (cm) used to calculate conservation scores. Black arrowheads point to mainly conserved residues in interface D (left) and E (right). The empty arrowhead points to a less conserved residue in interface E. (**b**–**e**) Conservation scores from sequence alignments (human, rat, chick, frog, fish and shark) of all Unc5 homologues (**b**,**d**) or only Unc5D (**c**,**e**) were mapped onto the surface of Unc5D. Black: highly conserved; white: not conserved. In our crystal structures, Lphn3 Lec (yellow ribbons) binds Unc5D Ig1 and Ig2 domains (interface D, black arrow head). Interface D residues are conserved across all Unc5 homologues (**b**) and across just Unc5D species (**c**). FLRT2^LRR^ (dark red ribbons) binds Unc5D TSP1 (interface E, black arrow head). Interface E residues are less conserved across Unc5A–D (**d**) compared with Unc5D species alone (**e**), suggesting that Unc5D is the only Unc5 homologue forming this interface.

**Figure 8:**
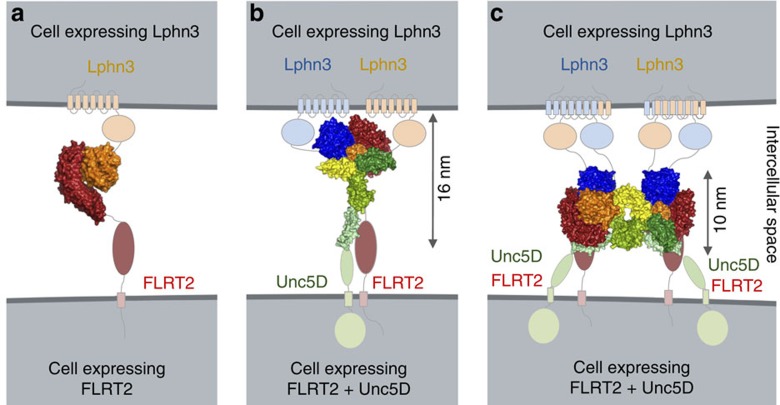
Arrangements of receptor super-complexes at the cell surface. (**a**) Current data support the idea that FLRT and Lphn engage in an adhesive in *trans* interaction across the synapse in a 1:1 stoichiometry. (**b**) 1:1:2 complexes, corresponding to one of the pseudo-symmetric halves in the complex structure of Unc5D^IgIgTSP^, FLRT2^LRR^ and Lphn3^Lec–Olf^, are presumably formed by all Unc5, FLRT and Latrophilin homologues. (**c**) Unc5D+FLRT2+Lphn3 form large (2:2:4) complexes in which two Unc5D ectodomains are arranged in an anti-parallel fashion. In our stripe assay, where Lphn3^Lec–Olf^ is presented in *trans* to Unc5D+FLRT2-expressing cells, the formation of this complex in *cis* would be non-adhesive.

Lphn3 also binds FLRT3 (refs 12, 13), the cognate interaction partner of Unc5B. It is therefore conceivable that Unc5B, and possibly other Unc5 homologues, could form super-complexes with FLRTs and Lphn3. We generated Unc5A–D sequence alignments (Fig. 7a) and used CONSURF^50^ to plot sequence conservation scores onto the surface of the Unc5D^IgIgTSP^ structure. The results show that the lectin-binding surface on Unc5D (interface D, Fig. 6c), which appears in the crystal structure of both the octamer and tetramer, is highly conserved across all Unc5 receptors (Fig. 7b,c). Conversely, the Unc5D TSP1 surface, which mediates binding to FLRT2^LRR^ and Unc5D Ig1 (interface E, Fig. 6b), is strongly conserved in Unc5D, but not across the other Unc5 receptor homologues (Fig. 7d,e). This analysis suggests that Unc5/FLRT/Lphn3 1:1:2 tetramer formation is likely conserved across Unc5 receptors, while only Unc5D may be able to further dimerize the complex to form an octamer via its TSP1 domain (Fig. 8).

Unc5D/FLRT2 functions are best understood with regard to cortical development in the mouse, where Unc5D is expressed in non-migrating neurons in the subventricular zone. Shed FLRT2 ectodomain diffusing from the cortical plate binds to Unc5D-expressing neurons, slowing down their migration towards the cortical plate. Conversely, FLRT3 is expressed in neurons in the intermediate zone, as they migrate towards the cortical plate which is rich in Unc5B (ref. 5). Latrophilins are broadly expressed in the mouse cortex^7^,^51^, and it is conceivable that the potentially different structural assemblies formed by Unc5D/FLRT2 and Unc5B/FLRT3 in the presence of Latrophilin reflect the different requirements of non-migrating versus migrating cells.

Taken together, our data reveal unexpected structural versatility for the interaction between three different types of cell surface receptors: the homophilic adhesion molecule FLRT; the repulsive guidance receptor Unc5; and the adhesion GPCR Latrophilin. We reveal a remarkable repertoire of structural assemblies is formed by these proteins, ranging from the previously described 1:1 dimers up to octameric super-complexes that bring together multiple copies of each protein. The results showcase how receptors increase their structural/functional versatility by engaging in different complexes, depending on the molecular make-up of their local environment.

## Methods

### Vectors and cloning

We cloned constructs of mouse Lphn3 (Q80TS3), mouse FLRT2 (UniProt Q8BLU) and rat Unc5D (UniProt F1LW30) into the Age1–Kpn1 or EcoR1–Kpn1 cloning site of vectors from the pHLSec family^52^, depending on whether the construct includes a native secretion signal sequence. For crystallization and biophysical experiments we cloned Unc5D^Ig^ (residues 1–161), Unc5D^IgIg^ (residues 1–244), Unc5D^IgIgTsp^ (residues 1–307), Unc5D^ecto^ (residues 1–382), Lphn3^Lec-Olf^ (residues 92–463), FLRT2^LRR^ (residues 35–362) and FLRT2^ecto^ (residues 35–540). For ITC experiments we cloned Lphn3^Olf^ (residues 199–495), and the entire extracellular domains of FLRT2 (residues 36–541) and human Unc5D (residues 32–383). For cell-binding assays, the mouse Latrophilin3 sequence, coding for the N-terminal fragment (residues 1–881), was cloned in pIgplus vector. For stripe assays, mouse FLRT2 (residues 35–660, wild type and H170N, UF) was fused to an N-terminal FLAG tag and a C-terminal Avitag and cloned into the pHLSec vector. Rat Unc5D (residues 46–956) was fused to an N-terminal HA tag and the pHLSec secretion signal and cloned into the pCAGIG vector (Unc5D-ires-GFP). For stripe assays with cells transfected with FLRT2 alone, mouse FLRT2 (residues 35–660) was fused to an N-terminal HA tag and the pHLSec secretion signal and cloned into the pCAGIG vector. For pull-down experiments, rat Unc5D (residues 46–956, wild type and W89N H91T, UF) was fused to an N-terminal HA tag and cloned into the AgeI–KpnI cloning sites of the pHLSec vector. This construct was fused to either a C-terminal mV tag (HA-Unc5D-mV) or Avitag, depending on the experiment. Murine Lphn3 (residues 20–1543, wild type and R292N R294T, LF) was cloned similarly, but was fused to an N-terminal Myc tag. For FLRT2, the N-terminally FLAG-tagged FLRT2 construct described above was used.

### Protein expression and purification

We expressed all proteins in either GlnTI-deficient HEK293S cells or kifunensine-treated HEK293T cells using established protocols^52^. Cell culture medium containing secreted recombinant proteins was clarified by centrifugation and filtration. Recombinant proteins were purified by Ni-affinity and size-exclusion chromatography. For ITC experiments, proteins were expressed as Fc fusions and affinity purified using Protein-A resin. Fc fusion proteins were cleaved using 3C protease to remove the Fc fragment, which was then separated by size-exclusion chromatography. The Lphn3-Fc chimera protein used in cell-binding assays was produced in HEK293 cells and purified by affinity chromatography (HiTrap Protein G HP column, GE Healthcare).

### Stripe assay

50 μg ml^−1^ Lphn3^Lec–Olf^ was mixed with 120 μg ml^−1^ Cy3-conjugated anti-Fc (Life Technologies A11014) in PBS. Matrices (90 μm width)^53^ were placed on 60-mm dishes and proteins injected. After 30 min incubation at 37 °C, dishes were washed with PBS and matrices removed. Dishes were coated with 50 μg ml^−1^ Fc protein mixed with 120 μg ml^−1^ anti-hFc (Jackson ImmunoResearch 109-005-098) for 30 min at 37 °C and washed with PBS. HeLa cells transfected with Unc5D-ires-GFP and/or FLAG-FLRT2 in pHLSec were cultured on the stripes for between 4 and 7 h, before fixing with 2% sucrose/4% paraformaldehyde in PBS for 10–20 min at room temperature. Wild-type and mutant FLRT2 constructs were expressed in HeLa cells on their own or together with Unc5D-ires-GFP, and stained using anti-FLAG (rabbit, Sigma F7425) and Alexa647-conjugated anti-rabbit (Life Technologies A21245) to ensure FLRT2 expression at the cell surface is not affected by the presence of Unc5D. Analysis was performed using ImageJ.

### Surface plasmon resonance

Equilibrium binding experiments were performed at 25 °C using a Biacore T200 instrument (GE Healthcare) using PBS+0.005% (v/v) polysorbate 20 (pH 7.5) as running buffer. The regeneration buffer was 2 M MgCl_2_. Unc5D^ecto^ was biotinylated enzymatically at a C-terminal Avitag and coupled to a streptavidin-coated CM5 chip. Data were analysed using the BIAevaluation software. *K*_d_ and *B*_max_ values were obtained by nonlinear curve fitting of a 1:1 Langmuir interaction model (bound=*B*_max_/(*K*_d_+*C*), where *C* is the analyte concentration calculated as monomer.

### Isothermal titration calorimetry

Experiments were performed with a MicroCal*iTC200* system. Protein solution in the syringe (100 μM) was added to the cell in a series of injections at 25 °C (injection volume varied from 1 to 1.5 μl). The concentration of protein in the MicroCal sample cell was 10 μM, with buffer alone in the reference cell. For the triple complex, FLRT2^ecto^+Lphn3^Olf^ were kept in the cell at 10 μM and Unc5D^ecto^ was injected at 100 μM. Raw ITC data were processed and fitted using a single-site model using the ORIGIN software provided by GE MicroCal, and the stoichiometry was not constrained during the model fitting. Blank experiments were performed in which concentrated protein was injected into the cell containing buffer alone. These experiments were subtracted from the positive data.

### Cell-binding assay

HEK293 cells were transfected with a pcDNA3 vector (Invitrogen) containing full-length mouse FLRT2 with a C-terminal FLAG tag. Fc (Jackson ImmunoResearch) and Unc5D^ecto^-Fc (R&D Systems) were pre-clustered with DyLight 594 Donkey Anti-Human IgG or Alexa Fluor 647 Donkey Anti-Human IgG, Fcγ Fragment Specific (both 1:400, Jackson ImmunoReserch, 709-515-098, 109-605-044) for 1 h at room temperature and were added to the cells at a concentration of 100 nM. Cells were incubated for 20 min at room temperature and washed, then Lphn3-Fc was added to the cells and incubated as before. Cells were fixed in 4% paraformaldehyde (10 min, room temperature) and permeabilized in PBS containing 0.1% Triton (Carl Roth) and 1% bovine serum albumin (Sigma) and immunostained for FLRT2 over-expression, with anti-FLAG antibody (1:1,000, Sigma F9291) and Alexa Fluor 488 Donkey Anti-Rabbit IgG (1:200, Jackson ImmunoReserch 711-545-152). Images were collected on a Leica SP8 microscope and quantification performed using ImageJ. For each image, the integrated density (the sum of the values of the pixels in the image) corresponding to bound protein was quantified and divided by the integrated density corresponding to FLRT2 over-expression.

### Pull-down assays

For Unc5D-mV pull downs, adherent HEK293T cells were transfected overnight with equal amounts of plasmids coding for mouse FLRT2 (N-terminal FLAG tag; C-terminal Avitag), mouse Lphn3 (N-terminal Myc tag; C-terminal Avitag) and wild-type or non-FLRT-binding (UF) rat Unc5D (N-terminal HA tag; C-terminal mV tag). For Lphn3-mV pull-downs, HEK293T cells were transfected overnight with equal amounts of plasmids coding for mouse FLRT2 (N-terminal Flag tag; C-terminal Avitag), Unc5D (N-terminal HA tag; C-terminal Avitag) and wild-type or non-FLRT-binding (LF) mouse Lphn3 (N-terminal Myc tag; C-terminal mV tag). The cells were washed with ice-cold PBS and resuspended in ice-cold lysis buffer (1% Triton X-100, 50 mM Tris-HCl (pH 7.5), 150 mM NaCl and protease inhibitors (EDTA-free, Sigma)). We disrupted the cells by mechanical force, incubated the lysates for 30 min on ice, removed cell debris by centrifugation and collected a first set of samples for analysis (input). Next, we added 2 μg ml^−1^ rabbit anti-GFP antibody (Life Technologies A11122) to the clarified lysate, incubated the lysate for 1 h at 4 °C, added 30 μl of protein G sepharose 4 FF (Sigma) per ml of lysate and incubated for a further 2 h at 4 °C. The lysate was removed and the sepharose washed twice with lysis buffer, once with lysis buffer and PBS mixed in a 1:1 volume ratio and once with PBS. The sepharose was boiled with SDS-containing loading buffer and bound proteins were revealed by western blot using mouse anti-Flag (1:1,000, Sigma F1804), mouse anti-HA (1:1,000, Sigma H3663) and chicken anti-Myc (1:1,000, AbCam ab19233) antibodies. Band intensities after pull down were measured using Image J and normalized within each blot. We plotted the normalized intensity ratios of anti-Myc/anti-HA (for Unc5D-mV pull down) or anti-HA/anti-Myc (for Lphn3-mV pull down) after dividing these ratios by the corresponding ratios in the ‘input' samples. Pull downs from brain lysate were prepared from cortex tissue (1-month-old mice) by homogenization in lysis buffer (50 mM Tris base, 150 mM NaCl, 1% NP-40 and protease inhibitors (Roche), pH 7.4). Samples were centrifuged at 1,000*g* for 10 min to discard tissue debris and nuclei, and the protein contents were determined by detergent-compatible protein assay (Bio-Rad, Hercules, CA). Immunoprecipitation was performed by incubation of 1.5 mg of total protein extracts with 1 μg of anti-Latrophilin3 antibody (Abcam ab140843) overnight at 4 °C followed by a 4-h incubation with 25 μl of protein G-Sepharose (GE Healthcare). The beads were washed by centrifugation three times and then boiled for 10 min in 7 μl of 6 × SDS sample buffer. The immunocomplexes were resolved on 7.5% SDS–PAGE and transferred to Immobilon-P transfer membrane. Blots were incubated with anti-Unc5D (1:1,000, Abcam ab58141) and detected using enhanced chemiluminescent reagents.

### Statistical analysis

Statistical analyses of stripe, pull-down and cell-binding assays were performed using GraphPad Prism, employing a two-tailed unpaired Student's *t*-test. *P≤0.05, **P≤0.01, ***P≤0.001 and ****P≤0.0001. The data are presented as the mean±s.e.m.

### Protein crystallography

Proteins for crystallization were purified as described above. Before size-exclusion chromatography, Lphn3^Lec–Olf^, FLRT2^LRR^ and Unc5D^Ig^ were incubated with recombinant endoglycosidase F1 (ref. 54) at room temperature (4–5 h) and then 4 °C overnight. Proteins were mixed in a 1:1:1 ratio and subjected to size-exclusion chromatography. The resultant peak fractions were pooled and concentrated to 13 mg ml^−1^ in 10 mM Tris-HCl (pH 8), 150 mM NaCl and 100 mM non-detergent sulfo-betaine (NDSB-256). Crystals were grown by the vapour diffusion method at 18 °C by mixing protein solution, crystallization solution and 2 mM L-arginine in a 1:1:1 ratio. The crystallization solution was 0.1 M sodium cacodylate (pH 6.5), 5% (w/v) poly-gamma-glutamic acid (PGA-LM) and 20% (w/v) polyethylene glycol 3350.

Lphn3^Lec–Olf^, FLRT2^LRR^ and Unc5D^IgIgTsp^ were deglycosylated at 4 °C overnight, subjected to size-exclusion chromatography, mixed in a 2:1:1 molar ratio and concentrated to 8 mg ml^−1^ in 10 mM Tris-HCl (pH 7.5), 150 mM NaCl and 100 mM NDSB-256. Crystals were grown by the vapour diffusion method at 18 °C by mixing protein and crystallization solution in a 1:1 (v/v) ratio for four separate crystallization solutions (crystallization solutions 1–4). Crystallization solution 1: 0.1 M MES (pH 6.0), 20% (v/v) 2-methyl-2,4-pentanediol (MPD), 2 mM HEPES (pH 6.8), 0.025% (w/v) benzidine, 0.025% (w/v) nicotinamide, 0.025% (w/v) pyromellitic acid and 0.025% (w/v) sulphaguanidine. Crystallization solution 2: 0.1 M MES (pH 6.0), 20% MPD, 2 mM HEPES (pH 6.8), 0.016% (w/v) 3-Indolebutyric acid, 0.016% (w/v) hexadecanedioic acid, 0.016% (w/v) oxamic acid, 0.016% (w/v) pyrometallic acid, 0.016% (w/v) sebacic acid and 0.016% (w/v) suberic acid. Crystallization solution 3: 0.1 M ammonium sulphate, 0.3 M sodium formate, 3% (w/v) poly-gamma-glutamic acid LM, 10% (w/v) polyethylene glycol monomethyl ether 2000 and 0.1 M sodium cacodylate (pH 6.5). Crystallization solution 4: 0.1 M MES (pH 6.0), 20% (v/v) MPD, 2 mM HEPES (pH 6.8), 0.02% (w/v) 2,5-pyridinedicarboxylic acid, 0.02% (w/v) pyromellitic acid, 0.02% (w/v) salicylic acid, 0.02% (w/v) *trans*-1,2-cyclohexanedicarboxylic acid and 0.02% (w/v) *trans*-Cinnamic acid.

### Structure determination

Crystals of the Lphn3^Lec–Olf^/FLRT2^LRR^/Unc5D^IgIgTSP^ complex were flash-frozen in a cryoprotectant solution containing 95% crystallization solution and 5% MPD. Diffraction data from four crystals were collected up to 3.4 Å resolution at the Diamond Light Source (beamline I04-1, *λ*=0.9281 Å) and the European Synchrotron Radiation Facility (ESRF; beamline ID29, *λ*=0.97625 Å) at 100 K. Data were integrated using XDS (via XIA2)^55^, and integrated intensities were merged, scaled and truncated using programmes from the CCP4 suite (BLEND)^56^. In choosing our highest-resolution cutoff we chose shells, which still fulfil CC1/2 (ref. 57) >25% and *I*/*σ*(*I*)⩾0.5 and which were supported by our results from the paired refinement method^57^. Choosing a lower resolution cutoff did not improve the overall quality of the maps. The structure was solved by molecular replacement in PHASER^58^, using the published higher-resolution structures of individual components, Lphn3^Lec–Olf^ and FLRT2^LRR^/Unc5D^Ig^ complex. A homology model of Unc5D^IgIgTSP^ (made with SWISS-MODEL^39^) based on the homologous structure of Unc5A^ecto^ (ref. 5) was placed manually into density that was clearly visible after the initial molecular replacement. The model was manually adjusted in COOT: most adjustments were required in loop regions of the Unc5D Ig2 and TSP1 domains, where the homology model was clearly not fitting the density, in the linker between the Lphn3 Olf and Lec domains, which adopts a different conformation in the complex compared with the unliganded Lphn3^Lec–Olf^ structure, and the Lphn3 DDD loop, which is not ordered in the unliganded structure. The model was all-atom refined in autoBUSTER^59^,^60^ without target, with the command line options -r 0.008 to restrain the geometry, -w 5 and AdjustXrayWeightAutomatically=no to fix the X-ray weight and -autoncs to use non-crystallographic symmetry restraints. Parameterization of thermal motion was based on results from the TLSMD web server^61^,^62^: each chain of Unc5D and Lphn3A was divided into two TLS bodies. The crystal of the Lphn3^Lec–Olf^/FLRT2^LRR^/Unc5D^Ig^ complex was flash-frozen in a cryoprotectant solution containing 75% reservoir solution and 25% glycerol. Diffraction data up to 6 Å resolution were collected at the Diamond Light Source (beamline I04-1, *λ*=0.9200 Å) at 100 K. The data were pseudo symmetric, and had to be processed by enforcing I4_1_22 as space group solution, using XIA2 (ref. 55). The structure was solved by molecular replacement in PHASER^58^ using components of the octameric structure described above, although the same solution is also found when using the previously published individual components FLRT2^LRR^, Lphn3^Lec–Olf^ and Unc5D^Ig^ (refs 5, 6). Manual assessment of the model was performed in COOT, but manual adjustment was not required^63^. The model was first rigid-body refined and then subjected to three cycles of all-atom refinement in autoBUSTER^59^,^60^ using the command line options -autoncs, -r 0.01 and by using the higher-resolution models of the individual components as targets^60^. During the first two cycles (100 small cycles each), the X-ray weight was maintained using the command line option -w 3 and AdjustXrayWeightAutomatically=no, for the last cycle (20 small), it was increased to -w10. Despite the overall modest resolution, the calculated electron density maps were of good quality due to the high multiplicity, high solvent content of the crystal and non-crystallographic symmetry (Supplementary Fig. 3). The quality of both final models was assessed using MolProbity^64^.

### Molecular dynamics simulations

Molecular dynamics simulations were performed with GROMACS 5.0 (ref. 65; www.gromacs.org) using the AMBER99SB forcefield^65^ with the ion modification provided by Joung and Cheatham^66^ in combination with the SPC/E water model. Structures were simulated with the Ca^2+^ and Na^+^ ions present in the central Lphn3^Olf^ channel in place. Na^+^ and Cl^−^ ions were added to a final concentration of 0.15 mM. Energy minimization was performed using the steepest descent algorithm and each system was equilibrated in a constant temperature (canonical example, NVT, 310 K) ensemble for 100 ps, followed by a 100ps equilibration at constant pressure (isothermal-isobaric, NPT, 1 bar). For equilibration and production runs, we used the velocity-rescaling thermostat, coupled separately for the protein and the solvent (ions and water) and the Parrinello–Rahman barostat, with a time constant of 2.0 ps and compressibility of 4.5 × 10^−5^ bar^−1^. During the equilibration phase, the non-hydrogen protein atoms were restrained by a force constant of 1,000 kJ mol^−1^ nm^−2^. Long-range electrostatics were modelled using the Particle-Mesh Ewald method. All bonds were treated using the LINCS algorithm. The integration time step was 2 fs. We then performed two different simulations: (i) 150 ns of unrestrained simulation to check the stability of the tetramer; and (ii) 70 ns of restrained simulation on the octamer structure. The restrained simulation was subsequently used to analyse the H-bonds formed by the residues at the protein interfaces. To analyse the hydrogen bond stability we used the VMD HBonds plugin (http://www.ks.uiuc.edu/Research/vmd/plugins/hbonds/) in combination with tcl in house scripts. Input parameters for VMD HBonds plugin were 60° and 3.6 Å for the cutoff angle and distance, respectively. We defined a stability value corresponding to the percentage of the simulation for which the residue (or atom) can form at least one H-bond with its partners.

### Mass spectrometry

Protein samples for MS were concentrated to 10–15 μM and buffer exchanged using dialysis into 0.5 M ammonium acetate at room temperature overnight or using micro Bio-Spin Columns (Bio-Rad). Immediately before MS analysis, the concentration of ammonium acetate was diluted to 0.2 M (pH 7.5). Native MS experiments were performed on a quadrupole-time-of-flight (Q-ToF) tandem mass spectrometer (Waters) previously modified for the transmission and detection of high molecular weight complexes^67^ and on a Synapt G1 mass spectrometer in time-of-flight-only mode (Waters). Protein solutions were introduced into the mass spectrometer using gold-coated capillary needles prepared in-house^68^. Typically, the following instrumental conditions were used for MS experiments: capillary voltage 1.5 kV, cone voltage 100–200 V and collision cell energy 20–50 V. For tandem MS experiments the collision energy was raised to 150 V. All mass spectra were calibrated off-line using a 10 mg ml^−1^ solution of cesium iodide.

### SEC-MALS

For all SEC-MALS experiments using Lphn3^Lec–Olf^, FLRT and Unc5 constructs, proteins were mixed in a 2:1:1 ratio and then concentrated to the desired concentration. Samples were loaded onto a Superdex 200 10/30 column (GE Healthcare) equilibrated in 10 mM Tris-HCl (pH 7.5) and 150 mM NaCl. The eluate was analysed using laser light scattering detected at 662 nm wavelength at eight scattering angles between 20.6° and 149.1° using a Heleos 8 instrument (Wyatt Technology, Germany). ASTRA 6.1 (Wyatt Technology) was used to calculate the molecular weights using the Zimm equation.

## Additional information

**Accession codes:** The RCSB PDB accession number for the tetrameric and octameric Lphn3:FLRT2:Unc5D complexes reported in this paper are 5ftu and 5ftt, respectively.

**How to cite this article:** Jackson, V. A. *et al*. Super-complexes of adhesion GPCRs and neural guidance receptors. *Nat. Commun.* 7:11184 doi: 10.1038/ncomms11184 (2016).

## Supplementary Material

Supplementary Information Supplementary Figures 1-7, Supplementary Table 1 and Supplementary Reference.

## Figures and Tables

**Table 1:** Crystallographic statistics.

**Data collection statistics**		
	**Lphn3**^**Lec–Olf**^**-FLRT2**^**LRR**^**-Unc5D**^**Ig**^	**Lphn3**^**Lec–Olf**^**-FLRT2**^**LRR**^**-Unc5D**^**IgIgTsp**^
PDB accession code	5ftu	5ftt
Space group	I 4_1_ 2 2	C 1 2 1
		
*Cell dimensions*		
*a*, *b*, *c* (Å)	293.00, 293.00, 291.72	231.96, 141.49, 151.49
*α*, *β*, *γ* (°)	90.00, 90.00, 90.00	90.00, 117.94, 90.00
Resolution (Å)	206.73–6.00 (6.34–6.00)	133.83–3.38 (3.47–3.38)
*R*_Meas_ (%)	21 (506)	15.1 (171.6)
*I/σI*	10.3 (0.6)	7.5 (0.5)
Highest resolution shell with *I/σI*>2	7.45–6.94	3.95–3.81
Completeness (%)	99.9 (99.7)	98.4 (92.4)
CC_1/2_	100 (26.3)	99.1 (39.2)
Multiplicity	13.3 (14.0)	8.0 (2.4)
		
*Refinement statistics*		
Resolution (Å)	206.73–6.01 (6.42–6.01)	133.83–3.4 (3.49–3.40)
No. of reflections	15,788 (2,411)	58,669 (2,832)
Clashscore, all atoms	3.32	3.51
*R*_work_*/R*_free_	0.276/0.278 (0.223/0.229)	0.225/0.245 (0.261/0.253)
		
*No. of atoms*		
Protein	25,542	19,496
Ligand/ion	138	120
		
*B*-factors		
Average *B*-factors	188	185
		
*R.m.s.d.s*		
Bond lengths (Å)	0.007	0.007
Bond angles (°)	0.86	0.92
		
*Ramachandran statistics*		
Most favoured regions (%)	91.6	91.1
Outliers (%)	0.67	0.29

Values in parentheses are for the highest-resolution shell. The clashscore is the number of serious steric overlaps (>0.4 Å) per 1,000 atoms^64^. Data from one crystal was used to solve the Lphn3^Lec–Olf^-FLRT2^LRR^-Unc5D^Ig^ structure. Data from four crystals were used to solve the Lphn3^Lec–Olf^-FLRT2^LRR^-Unc5D^IgIgTsp^ structure.
