# Supplementary material for: Super-complexes of adhesion GPCRs and neural guidance receptors
Source: Nat Commun. 2016 Apr 19;7:11184. doi: 10.1038/ncomms11184 (PMC4838878; doi:10.1038/ncomms11184)
Supplement: Supplementary Information — Supplementary Figures 1-7, Supplementary Table 1 and Supplementary Reference. [file ncomms11184-s1.pdf]

Supplementary Figures

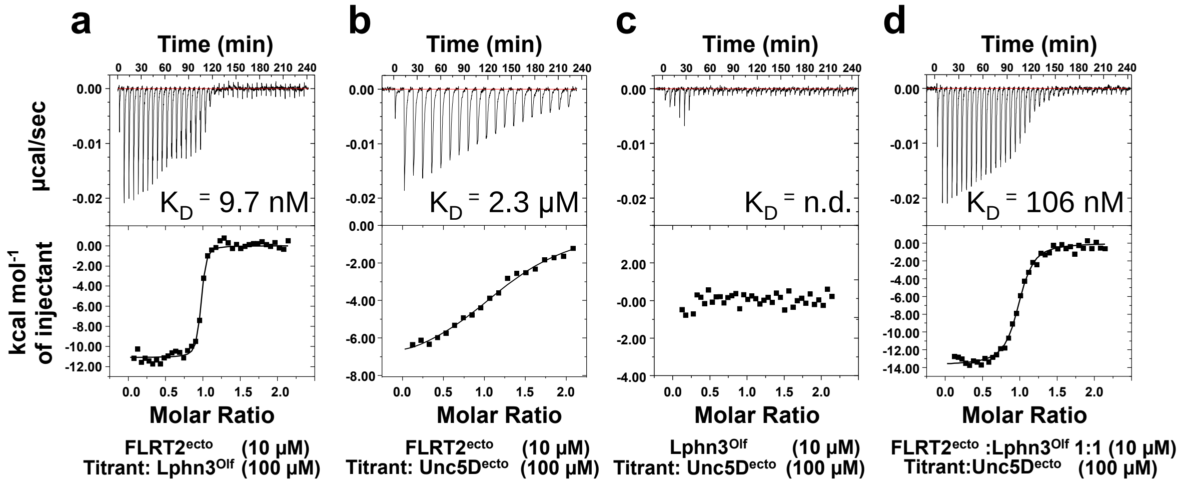

Supplementary Fig. 1. Isothermal Titration Calorimetry (ITC) data for each component of the ternary complex of FLRT2^{ecto}, Unc5D^{ecto} and Lphn3^{Olf}. a) FLRT2^{ecto} binding to Lphn3^{Olf}. **b)** FLRT2^{ecto} binding to Unc5D^{ecto}. **c)** Lphn3^{Olf} binding to Unc5D^{ecto}. **d)** FLRT2^{ecto} premixed with Lphn3^{Olf} binding to Unc5D^{ecto}. The data suggest a ~20-fold increase in Unc5D^{ecto} affinity for FLRT2^{ecto}+Lphn3^{Olf} compared to FLRT2^{ecto} alone.

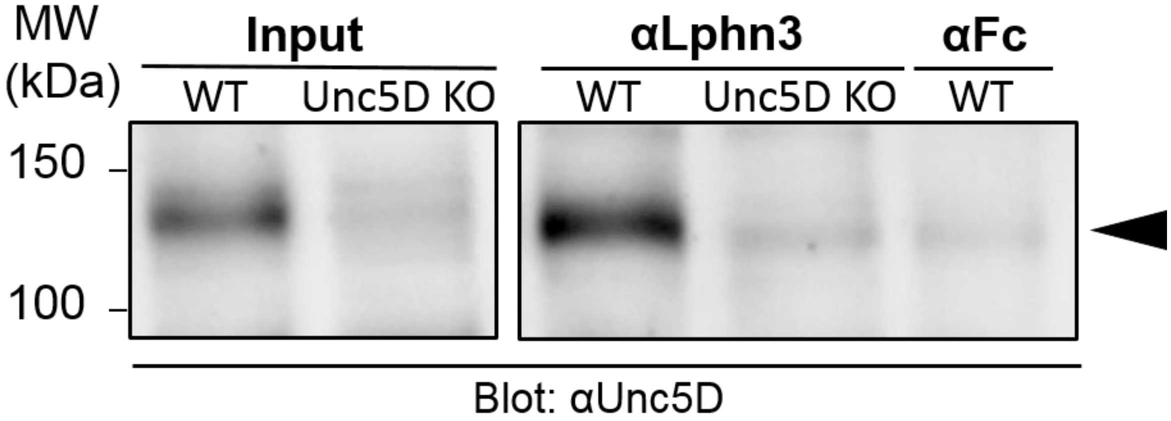

Supplementary Fig. 2. Unc5D is present in pulldowns of Lphn3 from cortical lysates. Anti-Lphn3 recognizing the C-terminus of murine Lphn3 was used to immobilise endogenous Lphn3 in cortical lysates of wild-type (WT) or Unc5D knockout (KO) adult mice. Fc instead of anti-Lphn3 was used as control for WT lysates. Blots were probed with anti-Unc5D antibody, revealing that Unc5D (black arrow head) is present in Lphn3 pulldown samples, but not in the controls.

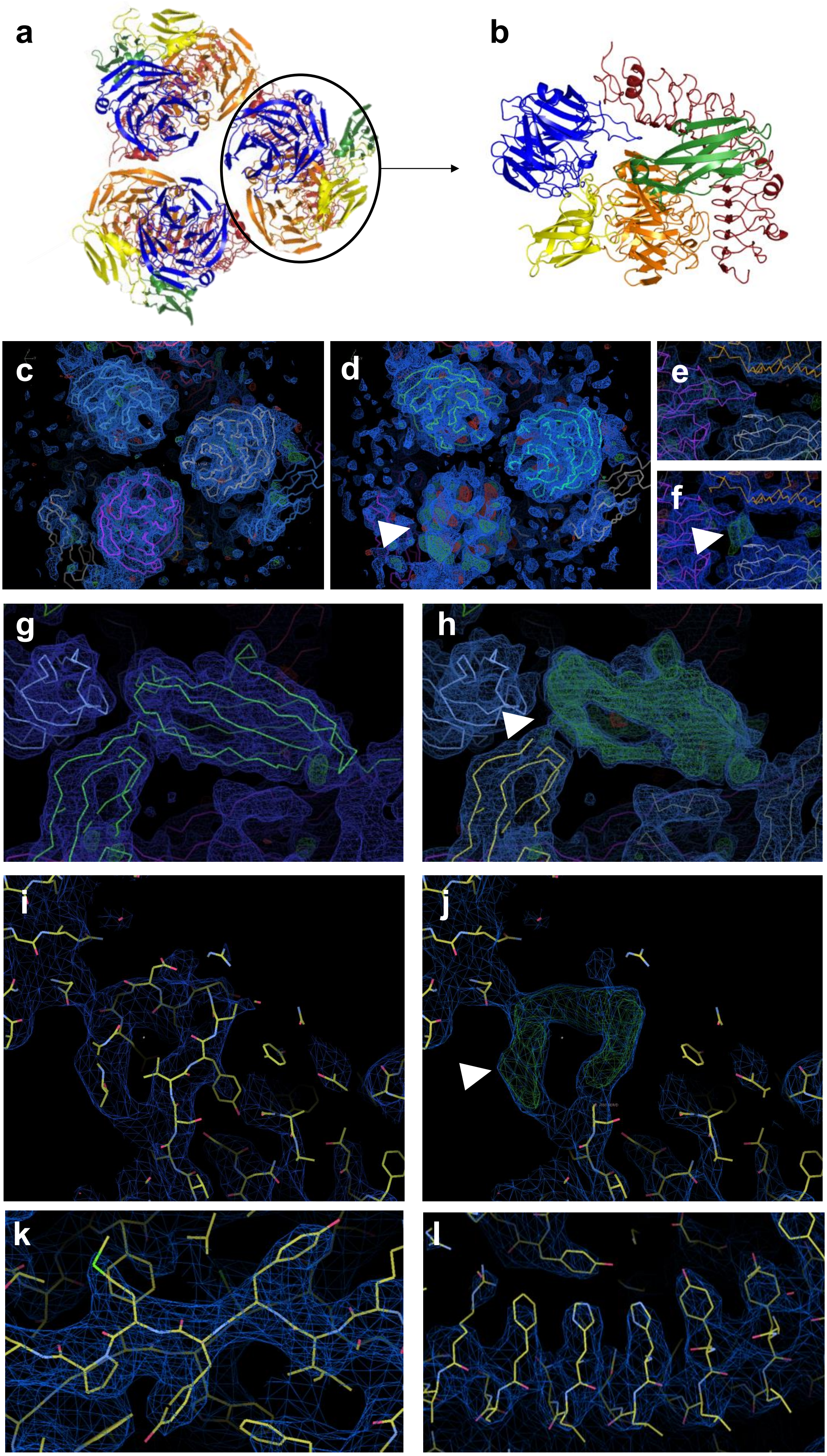
a
b
c
d
e
f
g
h
i
j
k
l

Supplementary Fig. 3. Crystal packing and electron density maps. a,b) The crystal structure of FLRT2^{LRR} and Unc5D^{Ig}, Lphn3$^{Lec\text{-}Olf}$ reveals three complexes in the asymmetric unit, each with the stoichiometry 1:1:2. The colours are orange (Olf domain of Lphn3A$^{Lec\text{-}Olf}$), yellow (Lec domain of Lphn3A$^{Lec\text{-}Olf}$), blue (Olf domain of Lphn3B$^{Lec\text{-}Olf}$), green (Unc5D^{Ig}), red (FLRT2^{LRR}). **c)** The X-ray diffraction data was integrated to 6 maximum Å resolution and resulted in good quality electron density maps. This allowed confident placement of the subunits, previously determined at higher resolution [1,2]. **d)** To validate the model, we generated maps after removal of individual chains. Here one Olf domain is removed (its previous location is highlighted by an arrow), resulting in strongly positive density for the missing chain. **e, f)**. As panels c and d, but focusing on the DDD-loop after removing it from the otherwise complete model. **g-j)** Electron density maps are shown as in previous panels, but here for data from the FLRT2^{LRR}/Unc5D^{IgIgTSP}, Lphn3$^{Lec\text{-}Olf}$ complex crystals. Panels g and i show maps calculated from the complete model, focusing on chain A (Unc5D Ig1) and chain D (Lphn3B DDD loop), respectively. Panels h and j show maps calculated after removal of modelled residues from these regions. **k,l)** Additional selected views of electron density maps derived from the FLRT2^{LRR}/Unc5D^{IgIgTSP}, Lphn3$^{Lec\text{-}Olf}$ complex data, focusing on the Lphn3 Olf domain (panel k) and the Lphn3^{Olf}/FLRT2^{LRR} interface (panel l). All 2Fo-Fc maps shown in the figure are visualised at the 1 sigma level in blue. Fo-Fc maps are shown at the 3 sigma level in red (negative) and green (positive).

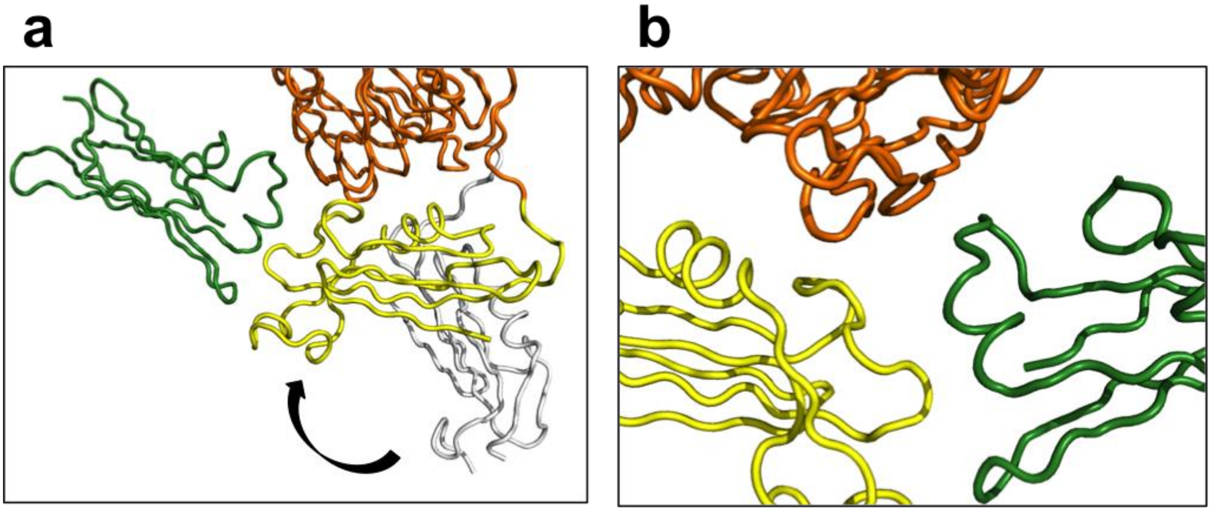

Supplementary Fig. 4. The Lphn3$^{Lec\text{-}Olf}$ linker region acts as a flexible hinge to reorientate the lectin domain. a) In the published structure of the unliganded Lphn3$^{Lec\text{-}Olf}$ the lectin and olfactomedin domains adopt an extended conformation (white). The lectin domain in the Lphn3$^{Lec\text{-}Olf}$:FLRT2^{LRR}:Unc5D^{Ig} structure (yellow) undergoes a rotation and translation. **b)** This movement brings the lectin domain into proximity with Unc5D^{Ig} and also with Lphn3^{Olf}.

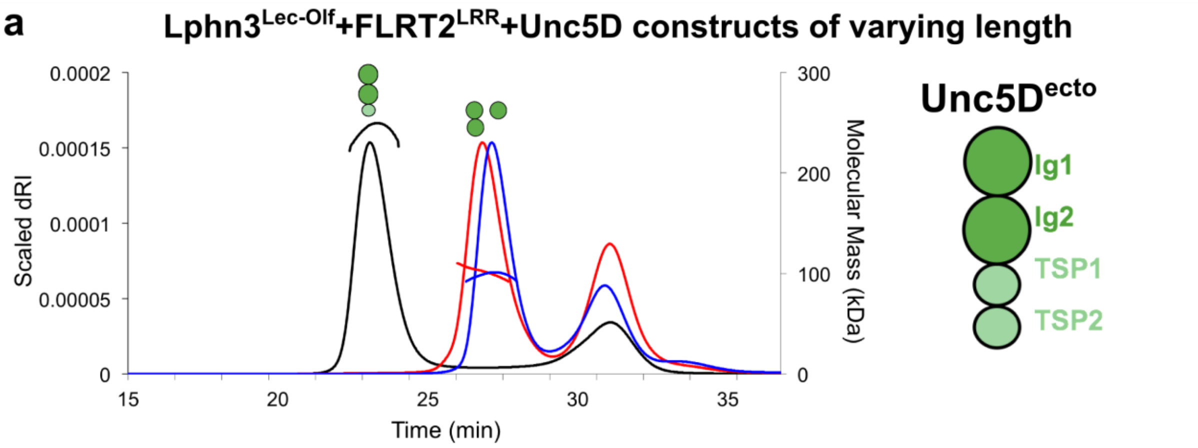

		Lphn3$^{Lec-Olf}$ + FLRT2^{LRR} +	Apparent MW (kDa)
		Unc5D^{Ig}	97 ± 0.339%
		Unc5D^{IgIg}	102 ± 0.308%
		Unc5D^{IgIgTSP}	243 ± 0.258%

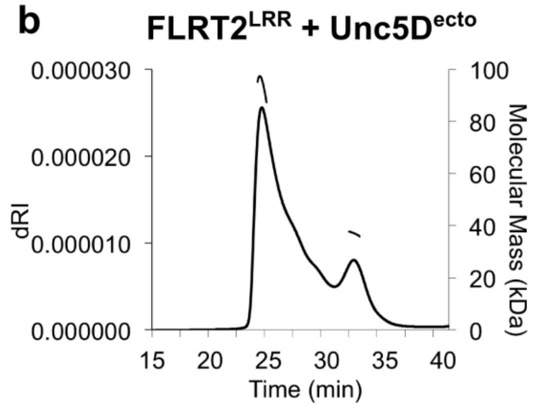

	Apparent MW (kDa)
FLRT2^{LRR} + Unc5D^{ecto}	94.3 ± 0.354%

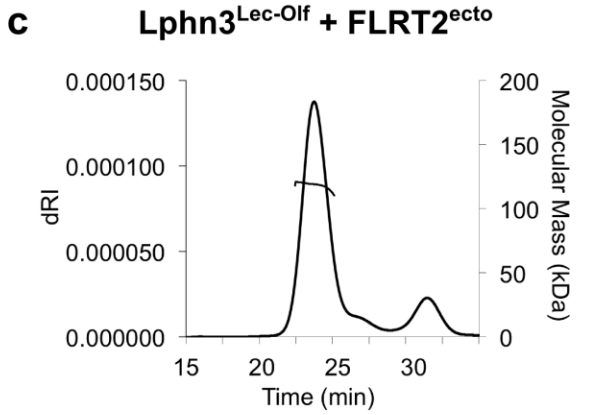

	Apparent MW (kDa)
Lphn3$^{Lec-Olf}$ + FLRT2^{ecto}	118.5 ± 0.254%

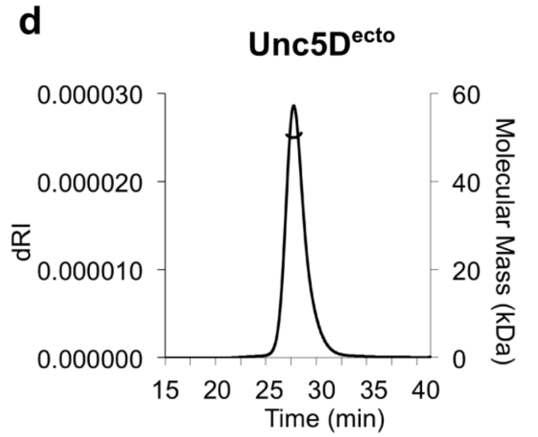

	Apparent MW (kDa)
Unc5D^{ecto}	50.2 ± 0.307%

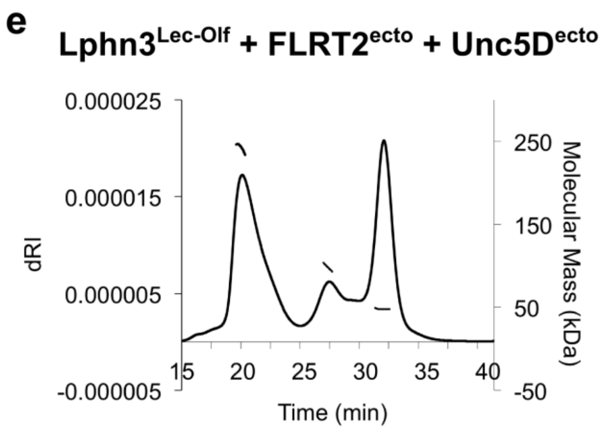

	Apparent MW (kDa)
Lphn3$^{Lec-Olf}$ + FLRT2^{ecto} +Unc5D^{ecto}	238.1 ± 0.308%

Supplementary Fig. 5. Size-exclusion chromatography coupled to Multi-angle light scattering (SEC-MALS). a) SEC-MALS indicates the formation of a large multimer (>>200 kDa) when Lphn3$^{\text{Lec-Olf}}$+FLRT2^{LRR} are mixed with Unc5D^{IgIgTsp}, but not when mixed with shorter Unc5D constructs without the TSP1 domain. For these experiments, complexes were injected at concentrations of 7 – 8 mg ml^{-1} (peak concentrations are ~0.7-0.8 mg ml^{-1}). **b,c)** Formation of the large complex depends on all three proteins being present, as FLRT2^{ecto}+Lphn3$^{\text{Lec-Olf}}$ or FLRT2^{LRR}+Unc5D^{ecto} form only 1:1 dimers on SEC-MALS. **d)** Unc5D^{ecto} alone does not oligomerise. **e)** Swapping FLRT2^{LRR} for FLRT2^{ecto} does not change or increase oligomerisation. Note that the measured samples are ~10-fold diluted by the SEC method, and therefore the peak concentrations reflect samples at low concentrations (<< 1 mg ml^{-1}). Protein complex formation is concentration-dependent, and so, at low concentrations, a fraction of the complex molecules separate into their subcomponents. The apparent molecular weight (MW) stated here reflects the average values within a given peak and so underestimates the true molecular weight of the fully formed complexes. We used native mass spectrometry to provide an accurate molar mass of the octameric complex (Fig. 5a).

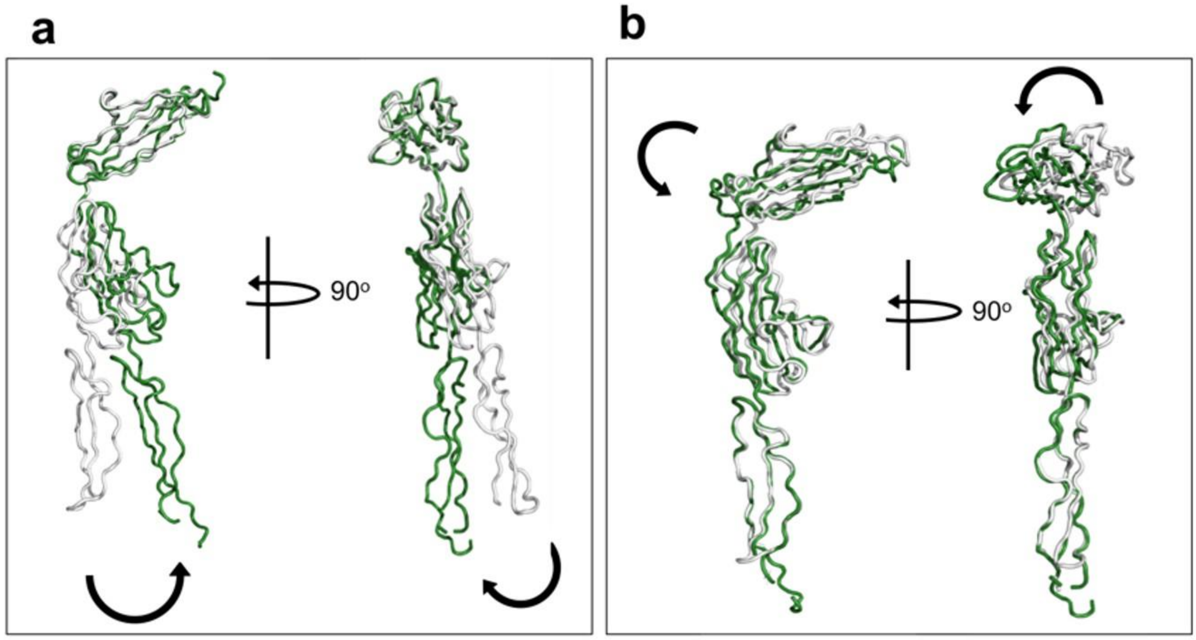

Supplementary Fig. 6. Comparison of Unc5A^{IgIgTSP} and Unc5D^{IgIgTSP} crystal structures. a, b) The structure of Unc5D^{IgIgTSP} (green) from the 4:2:2 Lphn3$^{Lec\text{-}Olf}$:FLRT2^{LRR}:Unc5D^{IgIgTsp} crystal structure was aligned with that of the unliganded Unc5A ectodomain (white, PDB accession number 4V2A)[1] via either the Ig1 domain (panel A) or the TSP1 domain (panel B), showing the major conformational difference lies within a hinge point in the Ig1-Ig2 linker, with a smaller reorientation around the Ig2-TSP1 linker.

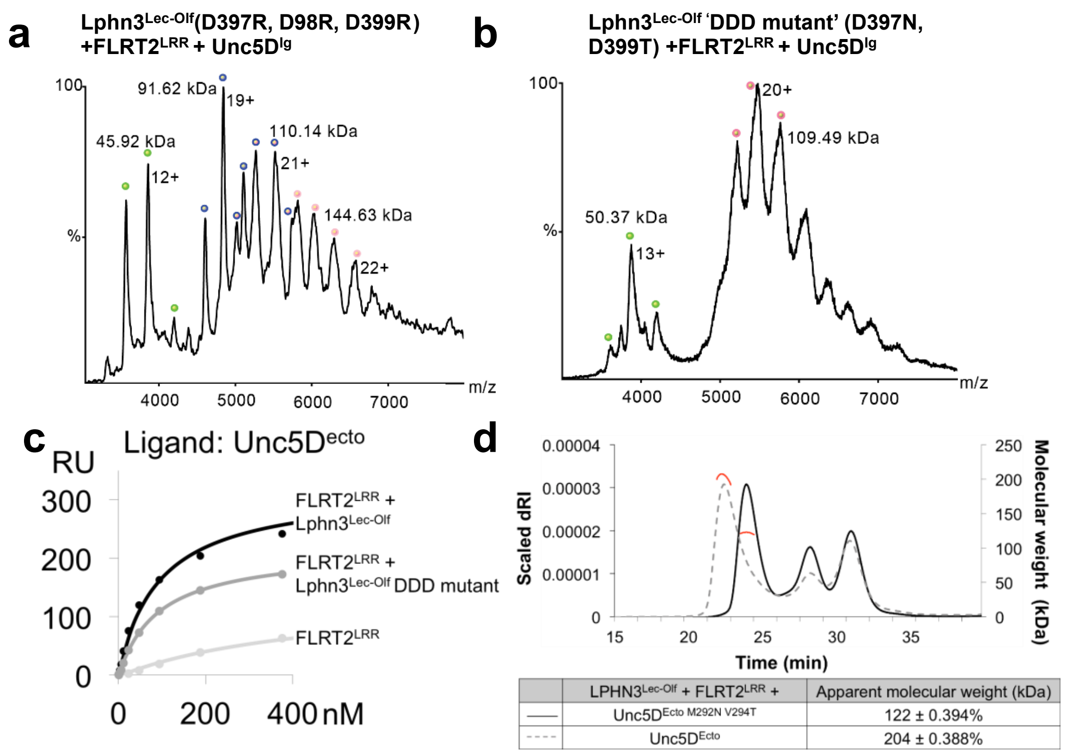

	LPHN3$^{Lec-Olf}$ + FLRT2^{LRR} +	Apparent molecular weight (kDa)
——	Unc5D$^{Ecto\ M292N\ V294T}$	122 ± 0.394%
- - -	Unc5D^{Ecto}	204 ± 0.388%

Supplementary Fig. 7. Site-directed mutagenesis disrupts interfaces B and E, resulting in smaller subcomplexes. a) Mass spectrometry shows that a charge swap in the DDD loop in Lphn3 reduces the presence of tetrameric complexes and increases the amount of 1:1:1 trimeric complex (compare with Fig. 4, panel a). The mass of the major species (~110 kDa) in the sample corresponds to a 1:1:1 trimer. **b)** Introduction of an N-linked glycan in the DDD loop fully disrupts the ability of Lphn3B to bind, resulting in only trimeric 1:1:1 complexes. **c)** The Lphn3$^{Lec-Olf}$ DDD mutant (mixed with FLRT2^{LRR}) displays an intermediate level of binding (RU_{max}) in surface plasmon resonance experiments using immobilised Unc5D^{ecto} when compared to wild-type Lphn3$^{Lec-Olf}$ + FLRT2^{LRR} protein or FLRT2^{LRR} alone. Note that the calculated K_Ds are similar for Unc5D^{ecto} binding to Lphn3$^{Lec-Olf}$ + FLRT2^{LRR} and Lphn3$^{Lec-Olf}$ DDD mutant + FLRT2^{LRR} (apparent K_D ~9 nM in both cases, compared to 460 nM for FLRT2 alone), suggesting that the enhanced affinity of FLRT2^{LRR} for Unc5D^{ecto} in the presence of Lphn3$^{Lec-Olf}$ depends mainly on Lphn3A. **d)** SEC-MALS indicates that the formation of large multimers, indicative of octamer formation (>>200 kDa, Supplementary Fig. 4a) of Lphn3$^{Lec-Olf}$+FLRT2^{LRR}

mixed with Unc5D^{ecto}, is abolished when using the Unc5D^{ecto} TSP1 mutant (M292N+V294T). Both protein samples were injected at ~3.5 mg ml^{-1}, resulting in ~0.3 mg ml^{-1} SEC peak concentrations. In contrast, using the Lphn3$^{Lec\text{-}Olf}$ DDD mutant and wild type Unc5D^{ecto} resulted in masses corresponding to a hexameric complex (not shown). As noted for Supplementary Figure 5, the apparent molecular weights stated here reflect the average values within a given peak and so can be used only for relative comparisons.

Supplementary Table 1:

interface	residue 1	residue 2	stability copy 1 (chains A,B,C,D)	stability copy 2 (chains E,F,G,H)
A	FLRT2 41 ARG	Lphn3A 350 ASN	0.48	NA
A	FLRT2 41 ARG	Lphn3A 347 GLU	NA	0.97
A	FLRT2 51 GLU	Lphn3A 294 ARG	1.00	1.00
A	FLRT2 52 ARG	Lphn3A 347 GLU	0.91	0.97
A	FLRT2 69 TYR	Lphn3A 319 ASP	0.98	0.97
A	FLRT2 69 TYR	Lphn3A 320 THR	0.91	0.86
A	FLRT2 69 TYR	Lphn3A 376 ARG	0.61	NA
A	FLRT2 71 HIS	Lphn3A 320 THR	0.98	0.97
A	FLRT2 72 ASN	Lphn3A 317 TYR	1.0	0.70
A	FLRT2 94 TYR	Lphn3A 319 ASP	0.77	0.74
A	FLRT2 96 TYR	Lphn3A 317 TYR	0.32	0.48
A	FLRT2 115 HIS	Lphn3A 245 TYR	0.72	0.63
A	FLRT2 115 HIS	Lphn3A 292 ARG	0.95	NA
A	FLRT2 115 HIS	Lphn3A 318 HIS	0.43	0.47
A	FLRT2 139 HIS	Lphn3A 245 TYR	0.77	0.87
A	FLRT2 139 HIS	Lphn3A 292 ARG	0.99	0.53
A	FLRT2 141 ASP	Lphn3A 292 ARG	0.96	0.91
A	FLRT2 186 ARG	Lphn3A 245 TYR	1.00	1.00
B	Lphn3B 397 ASP	Lphn3A 266 THR	1.00	0.97
B	Lphn3B 397 ASP	Lphn3A 304 ARG	0.82	0.57
B	Lphn3B 398 ASP	Lphn3A 265 THR	0.93	0.72
B	Lphn3B 398 ASP	Lphn3A 266 THR	0.33	NA
B	Lphn3B 400 ASN	Lphn3A 265 THR	0.51	0.73
B	Lphn3B 400 ASN	Lphn3A 267 THR	0.74	0.70
C	Lphn3B 292 ARG	Lphn3 152 TYR	0.91	0.81
C	Lphn3B 320 THR	Lphn3A 233 ALA	0.74	0.81
C	Lphn3B 376 ARG	Lphn3A 233 ALA	0.81	0.89
D	Unc5D 125 HIS	Lphn3A 306 LYS	0.69	0.76
D	Unc5D 125 HIS	Lphn3A 309 GLU	NA	0.51
D	Unc5D 127 PRO	Lphn3A 309 GLU	0.97	0.94
D	Unc5D 156 ARG	Lphn3A 105 GLU	0.9	NA
D	Unc5D 156 ARG	Lphn3A 182 TYR	0.59	NA
D	Unc5D 157 LYS	Lphn3A 135 ASP	0.88	0.95
D	Unc5D 157 LYS	Lphn3A 136 SER	0.50	0.43
D	Unc5D 238 ARG	Lphn3A 137 ASP	0.96	0.92

E*	Unc5D 272 LYS	FLRT2 102 GLU	0.98	0.96
E*	Unc5D 293 SER	FLRT2 101 ASP	0.99	0.62
E*	Unc5D 294 VAL	FLRT2 101 ASP	0.46	0.57
E*	Unc5D 272 LYS	FLRT2 101 ASP	NA	0.43
E*	Unc5D 296 LYS	FLRT2 123 THR	0.88	0.73
E*	Unc5D 49 GLY	Unc5D 297 ILE	0.97	0.92
E*	Unc5D 49 GLY	Unc5D 298 THR	0.99	0.95
E*	Unc5D 50 THR	Unc5D 296 LYS	0.8	0.77
E*	Unc5D 51 LEU	Unc5D 295 GLN	NA	0.59
E*	Unc5D 51 LEU	Unc5D 296 LYS	0.63	0.84
E*	Unc5D 140 LEU	Unc5D 270 TRP	0.36	0.73
E*	Unc5D 140 LEU	Unc5D 296 LYS	0.81	0.71
E*	Unc5D 140 LEU	Unc5D 297 ILE	0.57	0.62
E*	Unc5D 140 LEU	Unc5D 298 THR	NA	0.32
E*	Unc5D 142 THR	Unc5D 292 MET	0.41	0.36
E*	Unc5D 143 SER	Unc5D 292 MET	0.48	0.52
E*	Unc5D 146 ARG	Unc5D 290 GLU	0.63	0.76

* Values for copy 1 refer to binding of chain E to chains A and B. Values for copy 2 refer to binding of chain A to chains E and F.

Supplementary Table 1. Hydrogen bonds between interfacial residues in the FLRT2$^{Lec\text{-}Olf}$ + Unc5D^{IgIgTSP} + Lphn3$^{Lec\text{-}Olf}$ (2:2:4) complex. Residues, which form hydrogen bonds for at least 30% of the simulation time, are listed. Those forming bonds for at least 70% of the simulation time in both copies within the pseudo symmetric complex are highlighted in orange.

Supplementary Reference:

1. Seiradake, E. *et al.* FLRT Structure: Balancing Repulsion and Cell Adhesion in Cortical and Vascular Development. *Neuron* **84,** 370–385 (2014).
